# TET3 is a common epigenetic immunomodulator of pathogenic macrophages

**DOI:** 10.1172/JCI194879

**Published:** 2025-08-12

**Authors:** Beibei Liu, Yangyang Dai, Zixin Wang, Jiahui Song, Yushu Du, Haining Lv, Stefania Bellone, Yang-Hartwich Yang, Andrew Kennedy, Songying Zhang, Muthukumaran Venkatachalapathy, Yulia Surovtseva, Penghua Wang, Gordon G. Carmichael, Hugh S. Taylor, Xuchen Zhang, Da Li, Yingqun Huang

**Affiliations:** 1 Department of Obstetrics, Gynecology and Reproductive Sciences, Yale University School of Medicine, New Haven, Connecticut, USA.; 2 Center of Reproductive Medicine, National Health Commission Key Laboratory of Advanced Reproductive Medicine and Fertility, Shengjing Hospital of China Medical University, Shenyang, China.; 3 Assisted Reproduction Unit, Department of Obstetrics and Gynecology, Sir Run Run Shaw Hospital, School of Medicine, Zhejiang University, Hangzhou, China.; 4 Department of Metabolic Surgery, Jinshazhou Hospital of Guangzhou University of Chinese Medicine, Guangzhou, China.; 5 Department of Obstetrics and Gynecology, Shengjing Hospital of China Medical University, Shenyang, China.; 6 Center for Reproductive Medicine and Obstetrics and Gynecology, Affiliated Hospital of Medical School, Nanjing University, Nanjing, China.; 7 University of Virginia, Charlottesville, Virginia, USA.; 8 Department of Chemistry, Yale University, New Haven, Connecticut, USA.; 9 Yale Center for Molecular Discovery, Wester Haven, Connecticut, USA.; 10 Department of Immunology, University of Connecticut Health Center, Farmington, Connecticut, USA.; 11 Department of Genetics and Genome Sciences, University of Connecticut Health Center, Farmington, Connecticut, USA.; 12 Department of Pathology, and; 13 Yale Center for Molecular and Systems Metabolism, Yale University School of Medicine, New Haven, Connecticut, USA.; 14 Yale Liver Center, Yale University School of Medicine, New Haven, Connecticut, USA.

**Keywords:** Hepatology, Immunology, Inflammation, Epigenetics, Immunotherapy, Macrophages

## Abstract

Through a combination of single-cell/single-nucleus RNA-Seq (sc/snRNA-Seq) data analysis, immunohistochemistry, and primary macrophage studies, we have identified pathogenic macrophages characterized by Tet methylcytosine dioxygenase 3 (TET3) overexpression (Toe-Macs) in 3 major human diseases associated with chronic inflammation: metabolic dysfunction–associated steatohepatitis (MASH), non–small cell lung cancer (NSCLC), and endometriosis. These macrophages are induced by common factors present in the disease microenvironment (DME). Crucially, the universal reliance on TET3 overexpression among these macrophages enabled their selective elimination as a single population, irrespective of heterogeneity in other molecular markers. In mice, depleting these macrophages via myeloid-specific *Tet3* KO markedly mitigated disease progression, and the therapeutic effects were recapitulated pharmacologically using a TET3-specific small-molecule degrader. Through an unexpected mode of action, TET3 epigenetically regulated the expression of multiple genes key to the generation and maintenance of an inflammatory/immunosuppressive DME. We propose that Toe-Macs are a unifying feature of pathogenic macrophages that could be therapeutically targeted to treat MASH, NSCLC, endometriosis, and potentially other chronic inflammatory diseases.

## Abstract 2

Pathogenic TET3-overexpressing macrophges (Toe-Macs) across multiple inflammatory diseases can be selectively eliminated using a molecular glue-like molecule.

## Introduction

Macrophages play essential roles in maintaining tissue homeostasis and act as the first line of defense against pathogens. During disease, macrophages can be “educated” by the disease microenvironment (DME) to become phenotypically and functionally distinct disease-associated macrophages (DAMs), some of which accelerate disease progression (1). The nucleotide-binding domain, leucine-rich repeat and pyrin domain–containing 3 (NLRP3) inflammasome is an intracellular multiprotein complex that assembles in response to danger signals such as damage-associated molecular patterns (DAMPs) and pathogen-associated molecular patterns (PAMPs), resulting in host defense responses through cytokine production and inflammatory cell death, known as pyroptosis (2, 3). Under resting conditions, macrophages express NLRP3 at a low level that is not sufficient to initiate inflammasome formation. Usually, NLRP3 activation requires 2 sequential steps. The first step (signal 1) is “priming,” which is initiated primarily by DAMPs and PAMPs and leads to NF-κB–mediated transcriptional upregulation of NLRP3 and pro–IL-1β. The second step (signal 2) is NLRP3 oligomerization and inflammasome assembly triggered by diverse stimuli. The assembled complex acts as a platform for caspase-1 activation, maturation and release of IL-1β, and induction of pyroptosis (2, 3). In macrophages, the limiting step for NLRP3 activation is the transcriptional upregulation of NLRP3 (2, 3). Aberrant NLRP3 activation has been closely associated with a wide range of human disorders, including cardiovascular disease, neurodegeneration, metabolic dysfunction–associated steatohepatitis (MASH), non–small cell lung cancer (NSCLC), and endometriosis (4–8). However, modulating NLRP3 therapeutically has remained challenging due to our incomplete molecular understanding of its regulation, especially at the epigenetic level.

Tet methylcytosine dioxygenase 3 (TET3) belongs to the 3-member TET family dioxygenases, which also include TET2 and TET1. TET proteins function by oxidizing 5-methylcytosine (5mC) to 5-hydroxymethylcytosine (5hmC) to mediate DNA demethylation. TET proteins can also function by directly recruiting chromatin-modifying complexes independent of their catalytic activity (9). Clonal hematopoiesis of indeterminate potential (CHIP) is a common age-related phenomenon associated with increased risk of chronic liver disease and cardiovascular disease (4, 7, 8). In seminal studies involving TET2 deficiency, CHIP-associated MASH, atherosclerosis, and cardiac arrhythmias, an unconstrained inflammatory response from TET2-deficient macrophages has been implicated in disease pathophysiology (4, 7, 8). These TET2-deficient macrophages displayed elevated NLRP3 activity with increased secretion of proinflammatory cytokines such as IL-1β and IL-6 (4, 7, 8). Intriguingly, in TET2-deficient macrophages the expression of TET3 was increased relative to that in WT macrophages (GSE223694) (7), leaving unresolved the possibility that TET3 contributes to the hyperactivity of NLRP3 in these diseases.

MASH is a chronic liver disease characterized by excessive hepatic lipid accumulation, inflammation, and hepatocyte damage with varying degrees of fibrosis and is a fast-growing chronic liver disease with limited therapeutic options (10). Emerging evidence suggests an important role for NLRP3 dysfunction in MASH pathophysiology (7, 11). In CHIP-associated MASH, hyperactive macrophage NLRP3 has been linked to increased liver inflammation and fibrogenesis (7). When challenged with a MASH-inducing diet, mice with *Nlrp3* deletion specifically in the myeloid compartment showed a significant decrease in hepatic inflammation and fibrotic changes (12). Thus, both human and mouse studies have pinpointed myeloid NLRP3 dysfunction as a key driver of hepatic inflammation and fibrosis. Yet, the molecular underpinnings of NLRP3 dysfunction have remained elusive.

NSCLC is one of the most lethal cancer types responsible for the majority of cancer-related deaths worldwide. Recent immunotherapy using immune checkpoint inhibitors (ICIs) has revolutionized cancer treatment, but these ICIs elicit beneficial effects only in a minority of patients; the major mechanism limiting their efficacy is an inflammatory, immunosuppressive tumor microenvironment (TME) with tumor-associated macrophages (TAMs) being the main culprits (13, 14). Aberrant NLRP3 activation is associated with various types of human cancer; its detrimental effects are attributed mainly to its effector cytokine IL-1β (5). Indeed, a causal role of IL-1β in NSCLC has been implicated from a phase III clinical trial showing that treatment with canakinumab, a human monoclonal antibody specific for IL-1β, reduced NSCLC incidence and lung cancer mortality in a dose-dependent manner (15).

Endometriosis is a systemic chronic inflammatory disease defined as the growth of endometrium-like tissue outside the uterus (16). Roughly 10% of women of reproductive age worldwide are affected by endometriosis, which causes pain and infertility (16). Macrophages are among the most abundant immune cells present in endometriotic lesions and play a central role in the growth, vascularization, and innervation of lesions as well as the generation of pain symptoms (17). Treating mice with MCC950, a small-molecule NLRP3-specific inhibitor, reduced endometriosis lesion size, suggesting a pathogenic role of NLRP3 hyperactivation in endometriosis (6).

Despite the unequivocal role of DAMs in the pathophysiology of MASH, NSCLC, and endometriosis, therapeutic targeting of DAMs has remained extremely challenging because these cells are phenotypically and functionally heterogenous and because DAMs with either disease-promoting (pathogenic) or disease-inhibiting (protective) properties coexist in the DME (13, 14). The identification of molecular features specifically associated with pathogenic macrophages and the development of strategies enabling the selective elimination of these macrophages are of paramount importance.

In the current work, we identify pathogenic macrophages characterized by TET3 overexpression (herein referred to as Toe-Macs) in MASH, NSCLC, and endometriosis. While not identical in other molecular features and surface markers, these macrophages share the hallmark of TET3 overexpression–induced hyperactivity of multiple genes known to play critical roles in the generation and maintenance of an inflammatory and/or immunosuppressive DME. We provide evidence that these macrophages are induced by the DME and represent a unifying feature of pathogenic macrophages that can be selectively depleted as a single population owing to shared vulnerability to TET3 reduction.

## Results

### Toe-Macs are present in human MASH livers.

**Figure 1:**
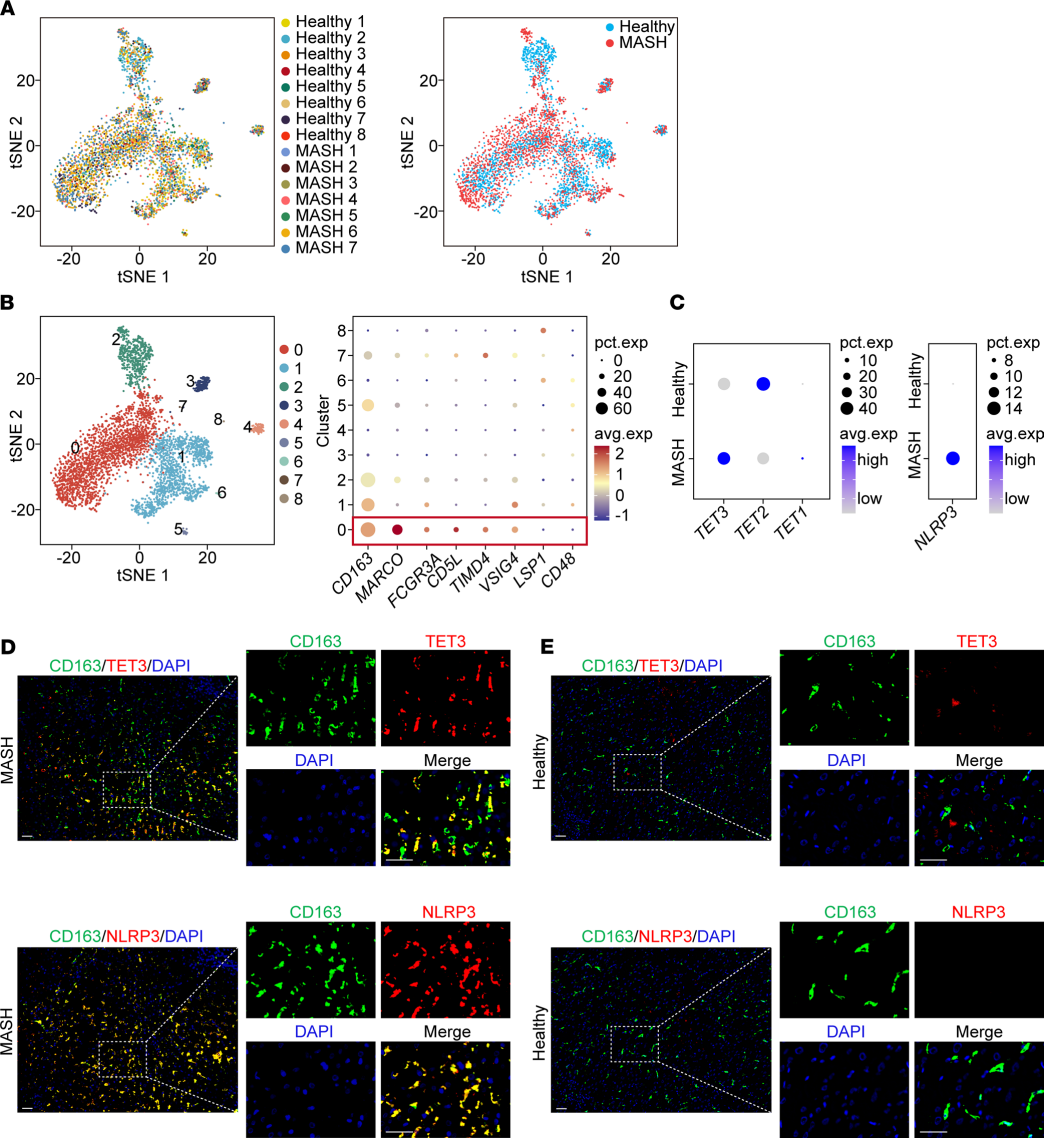
TET3- and NLRP3-overexpressing macrophages in human MASH liver. (**A**) Dimension reduction plots showing all myeloid cells from healthy and MASH livers. (**B**) Dimension reduction plot showing 9 clusters of total myeloid cells and a dot plot indicating that cluster 0 expresses classical KC markers. pct.exp, percentage of expression; avg.exp, average expression. (**C**) Dot plots showing expression levels of *TET3*, *TET2*, *TET1* (left panel), and *NLRP3* (right panel) in KCs. (**D**) IHC images of TET3 (red, top panels) and NLRP3 (red, bottom panels) costaining with CD163 (green) and DAPI (nuclei, blue) in liver tissue sections from MASH. The top panels are serial sections of the bottom panels. (**E**) IHC images of TET3 and NLRP3 costaining with CD163 and DAPI (nuclei) of liver tissue sections from healthy controls. The top panels are serial sections of the bottom panels. Scale bars: 10 μm. *t*SNE, *t*-distributed stochastic neighbor embedding. Refer to Supplemental Table 1 for detailed patient sample information.

Liver macrophages have 2 origins: embryonically derived resident tissue macrophages (RTMs) (Kupffer cells [KCs]) and monocyte-derived macrophages (mo-Macs). At homeostasis, the KC population is maintained through self-renewal. During MASH, bone marrow–derived monocytes infiltrate the liver, gradually lose expression of monocyte genes, acquire KC characteristics, and dominate the KC pool (18, 19). We analyzed publicly available single-nucleus RNA-Seq (snRNA-Seq) datasets derived from liver tissue samples of patients with MASH and healthy controls (20). Figure 1A shows all myeloid cells from healthy and MASH livers. Unsupervised clustering identified 9 clusters of myeloid cells, with cluster 0 consisting predominantly of KCs based on 6 positive (including CD163) and 2 negative KC markers (Figure 1B). Interestingly, KCs in MASH livers predominantly overexpressed TET3 (Toe-Macs), while those in healthy livers primarily expressed high levels of TET2 (Figure 1C, left panel). TET1 expression was minimal, which aligns with previous findings that TET2 and TET3 are the main TET isoforms in macrophages (21). NLRP3-overexpressing (NLRP3-oe) KCs also largely appeared in MASH livers (Figure 1C, right panel). In contrast to KCs, other myeloid cells (non-KCs, clusters 1–8) with high TET3 expression were more common in healthy livers (Supplemental Figure 1; supplemental material available online with this article; https://doi.org/10.1172/JCI194879DS1). Using IHC analysis of serial liver sections, we detected Toe-Macs and NLRP3-oe Macs in MASH, but not in healthy, livers (Figure 1, D and E, and Supplemental Figure 2). We observed heterogenous subcellular localization of TET3 in macrophages (Figure 1D and Supplemental Figure 2A), consistent with our previous report (22). These results suggest that Toe-Macs are pathogenic and induced by the DME, and that TET3 regulates NLRP3 expression within these cells.

### Toe-Macs are likely induced by the DME via a positive feedback loop.

**Figure 2:**
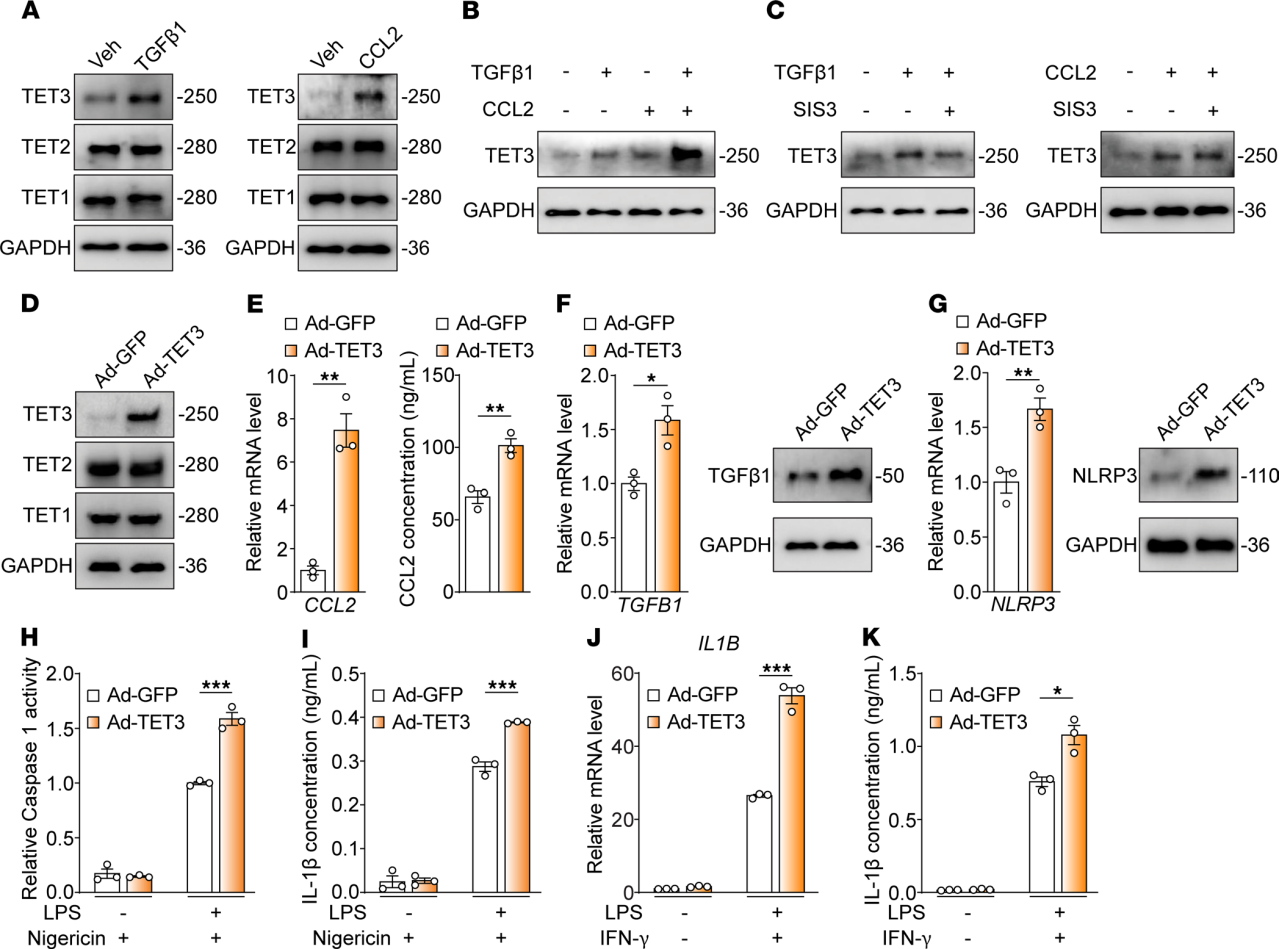
Toe-Macs induced by DME factors exhibit elevated NLRP3 activity. (**A**) MDMs were treated with Veh, TGF-β1 (10 ng/mL), or CCL2 (200 ng/mL). Proteins were isolated at 24 hours (Veh/TGFβ1) or 48 hours (Veh/CCL2) for Western blot analysis. Protein sizes are in indicated in kDa. (**B**) MDMs were treated with Veh, TGF-β1 (10 ng/mL), CCL2 (200 ng/mL), or TGF-β1 (10 ng/mL) plus CCL2 (200 ng/mL). Proteins were isolated at 48 hours. (**C**) MDMs were treated with Veh, TGF-β1 (10 ng/mL), or CCL2 (200 ng/mL), with or without SIS3 (a SMAD3 inhibitor) (5 mM). Proteins were isolated at 48 hours. (**D**) Western blot analysis of MDMs infected with Ad-GFP or Ad-TET3 for 24 hours. (**E**) *CCL2* expression in MDMs assessed by qRT-PCR (RNA harvested at 24 hours) and ELISA (supernatants harvested at 48 hours). (**F**) *TGFB1* expression in human MDMs assessed by qRT-PCR (RNA harvested at 24 hours) and Western blotting (protein harvested at 48 hours). (**G**) *NLRP3* expression in MDMs assessed by qRT-PCR (RNA harvested at 12 hours) and Western blotting (protein harvested at 24 hours). (**H** and **I**) MDMs seeded in 96-well plates were infected with Ad-GFP or Ad-TET3 for 24 hours. Cells were primed with or without LPS at 250 ng/mL for 4 hours. Nigericin was added at 20 mM for 1 hour, followed by measurement of caspase-1 activity (**H**) and IL-1β protein levels (**I**). (**J**) MDMs were infected with Ad-GFP or Ad-TET3 for 24 hours prior to stimulation with 10 ng/mL LPS plus 20 ng/mL IFN-γ for 4 hours, followed by qRT-PCR of *IL1B* mRNA. (**K**) ELISA analysis (after 8 hours of LPS/IFN-γ stimulation) of IL-1β of MDMs treated as in **J**. All data represent the mean ± SEM. *P < 0.05, **P < 0.01, and *** *P* < 0.001, by 2-tailed Student’s *t* test. Western blot data are representative of 2–3 biological repeats.

We found that exposing human peripheral blood monocyte-derived macrophages (MDMs) to TGF-β1 or CCL2 significantly increased TET3 expression at both the mRNA and protein levels (22). This is particularly relevant, given that the MASH microenvironment often has elevated levels of these factors (23–25). Specifically, while TGF-β1 or CCL2 alone boosted TET3 protein levels without affecting TET2 or TET1 (Figure 2A), they also showed a synergistic effect on TET3 upregulation (Figure 2B). Similar results were seen in mouse bone marrow–derived macrophages (BMDMs) (Supplemental Figure 3A). Interestingly, blocking Smad3-mediated TGF-β1 signaling inhibited the TGF-β1–induced TET3 increase but did not affect the CCL2-stimulated increase (Figure 2C). These findings support the idea that factors within the DME induce Toe-Macs.

During liver inflammation and fibrosis, mo-Macs are primarily drawn to the inflamed liver by the CCL2/CCR2 signaling pathway (26). Cenicriviroc (CVC), a dual CCR2/CCR5 antagonist, effectively reduced MASH fibrosis in a phase IIb clinical trial (27). Additionally, liver macrophage-derived TGF-β1 promotes fibrotic MASH (23). As both CCL2 and TGF-β1 are secreted proteins capable of inducing TET3 overexpression (Figure 2, A–C, and Supplemental Figure 3A), we hypothesized that TET3 promotes CCL2 and TGF-β1 expression in macrophages. To test this, TET3 expression was increased in human MDMs through infection with an adenoviral vector expressing a Flag-tagged human TET3 catalytic domain (Ad-TET3), or a control vector expressing GFP (Ad-GFP) (22). While increased TET3 expression did not alter the expression of TET2 or TET1 (Figure 2D), CCL2 expression was increased at both the mRNA and protein levels in Ad-TET3–infected compared with Ad-GFP–infected cells (Figure 2E). Similar observations were made for TGF-β1 (Figure 2F). We conclude that TET3 upregulates macrophage CCL2 and TGF-β1 expression likely through a positive feedback loop involving TET3, CCL2, and TGF-β1.

### TET3 overexpression sensitizes macrophages to NLRP3 activation and IL-1β production.

In MASH livers, various triggers, such as uric acid crystals, cholesterol crystals, and extracellular ATP, can induce intracellular change (like an increase in ROS, lysosomal destabilization, or potassium efflux) that promotes NLRP3 oligomerization and inflammasome assembly. Of these, potassium efflux is considered the common mechanism for all NLRP3 ligands in activating the inflammasome (11). When TET3 was overexpressed in human MDMs (Figure 2D), it increased NLRP3 expression at both the mRNA and protein levels (Figure 2G). Nigericin is a potassium ionophore that activates the NLRP3 inflammasome by promoting potassium efflux. When TET3-oe MDMs were stimulated with LPS followed by nigericin, we observed increased caspase-1 activity (Figure 2H) and IL-1β production (Figure 2I). We obtained similar results in mouse BMDMs (Supplemental Figure 3, B–H). These results suggest that TET3 overexpression rendered macrophages more susceptible to NLRP3 inflammasome activation. IFN-γ can synergize with LPS to increase the expression of NLRP3 and IL-1β and activates the alternative NLRP3 inflammasome in a K^+^ efflux-independent manner (2). Interestingly, TET3 also boosted IL-1β production in MDMs following stimulation with LPS and IFN-γ (Figure 2, J and K), indicating it might directly enhance IL-1β at the transcriptional level.

### TET3 regulates the expression of TGF-β1, NLRP3, IL-1β, and CCL2 at the epigenetic level.

**Figure 3:**
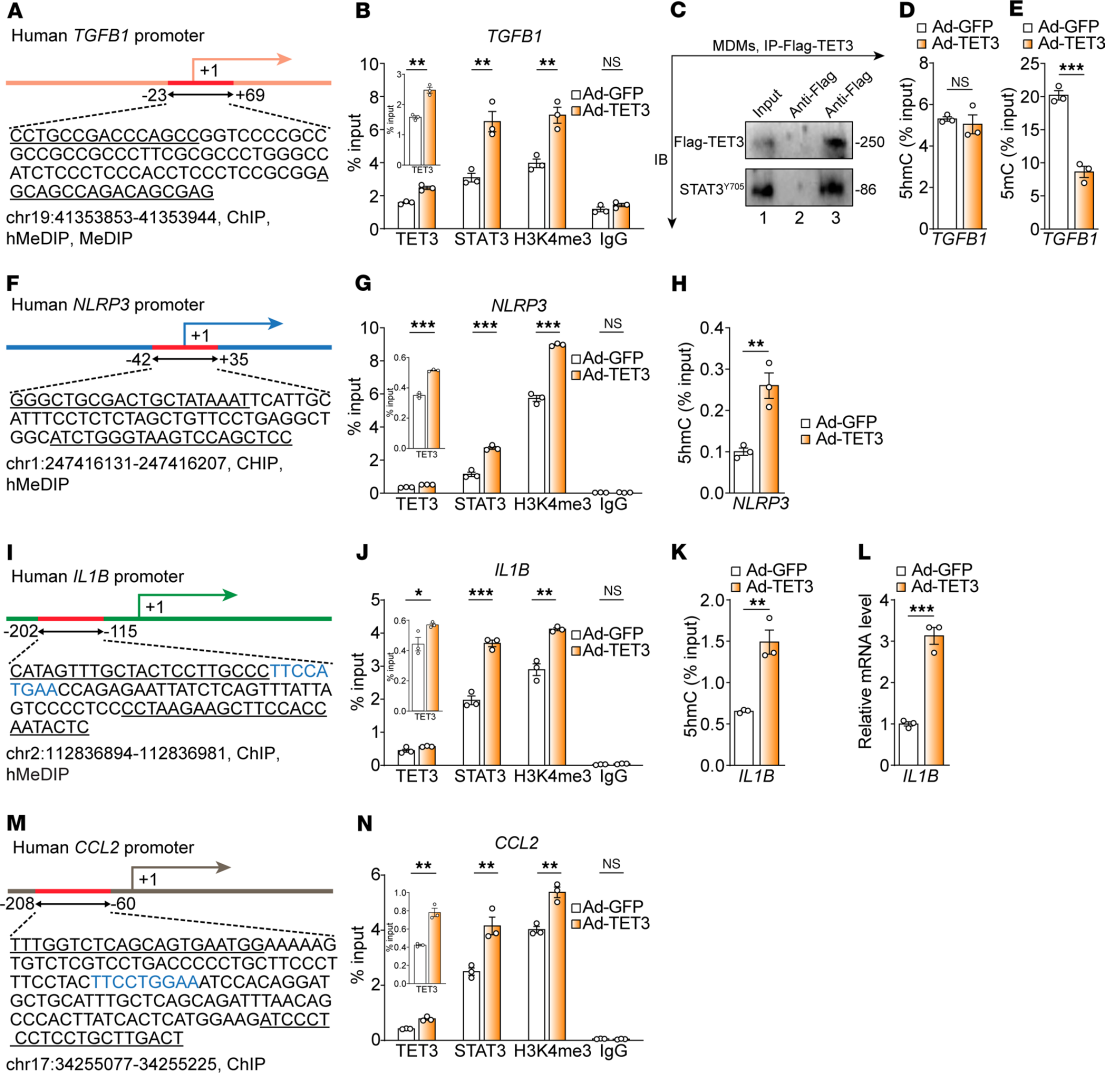
TET3 epigenetically regulates *TGFB1*, *NLRP3*, *IL1B*, and *CCL2* expression through interaction with phosphorylated STAT3. (**A**) Schematic of the human *TGFB1* promoter. Numbers depict nucleotide positions relative to the transcription start site labeled +1. The PCR-amplified region is marked in red, with the zoomed-in sequence shown underneath. The PCR primer sequences are underlined. (**B**) MDMs were infected with Ad-GFP or Ad-TET3 for 16 hours. Chromatins were prepared for ChIP-qPCR analysis to detect enrichment of the specific *TGFB1* promoter region outlined in **A**. (**C**) MDMs were infected with Ad-GFP (lane 2) or Ad-TET3 (lane 3) for 24 hours, followed by co-IP using anti-Flag antibody. Western blot analysis was carried out using anti-TET3 or anti-STAT3 (Y705) antibodies. Lane 1 shows 5% of input from Ad-TET3–infected cells. IB, immunoblot. (**D** and **E**) MDMs were treated as in **B**. Genomic DNA was collected and subjected to hMeDIP-qPCR (**D**) and MeDIP-qPCR (**E**). (**F**) Schematic of the human *NLRP3* promoter. (**G**) MDMs were treated as in **B**, followed by ChIP-qPCR. (**H**) MDMs were treated as in **B**, followed by hMeDIP-qPCR. (**I**) Schematic of the human *IL1B* promoter. The STAT3-binding site is highlighted blue. (**J**) MDMs were treated as in **B**, followed by ChIP-qPCR. (**K**) MDMs were treated as in **B**, followed by hMeDIP-qPCR. (**L**) Basal *IL1B* expression in MDMs infected with Ad-GFP or Ad-TET3 was assessed by qRT-PCR. (**M**) Schematic of the human *CCL2* promoter. The STAT3-binding site is highlighted blue. (**N**) MDMs were treated as in **B**, followed by ChIP-qPCR. In **B**, **G**, **J**, and **N**, 5 × 10^5^ cells per ChIP were used. All data represent the mean ± SEM. *P < 0.05, **P < 0.01, and ***P < 0.001, by 2-tailed Student’s *t* test. Western blot data are representative of 2 biological repeats. chr, chromosome.

DNA methylation (5mC) and hydroxymethylation (5hmC) are critical epigenetic modifications important for regulating gene expression. Typically, DNA methylation represses gene transcription, whereas 5hmC modification or demethylation enhances gene expression. TET proteins initiate DNA demethylation by oxidizing 5mC to 5hmC, with 5hmC also functioning as a stable epigenetic mark (9). Our previous studies demonstrated that TET3 expression is upregulated in human and mouse hepatocytes and hepatic stellate cells during liver fibrosis and that TET3 epigenetically enhances TGF-β1 expression (28, 29). Using genome-wide single-nucleotide resolution methylation analysis and ChIP followed by quantitative PCR (ChIP-qPCR), we identified a specific TET3-binding region in the human *TGFB1* promoter that underwent demethylation (Figure 3A). This demethylation event resulted in an open chromatin state marked by the histone H3 at lysine 4 trimethylation (H3K4me3) facilitating active transcription of *TGFB1* (28–30). However, how TET3, a broad DNA-binding protein, is recruited to this specific region of the *TGFB1* promoter remained unresolved in previous studies. Given our prior finding that TET3 modulates *AGRP* gene transcription in AgRP neurons via interaction with the transcription factor STAT3 (31), we hypothesized that TET3 overexpression in macrophages leads to STAT3-mediated recruitment of TET3 to the *TGFB1* promoter, facilitating 5hmC modification, subsequent demethylation, chromatin remodeling, and activation of *TGFB1* transcription. To investigate this, we infected human MDMs with Ad-TET3 or Ad-GFP and conducted ChIP-qPCR analyses. Compared with Ad-GFP–infected cells, Ad-TET3 infection significantly enhanced TET3, STAT3, and H3K4me3 occupancy at the *TGFB1* promoter (Figure 3B). When cytokine or growth factor receptors are activated, STAT3 is phosphorylated at tyrosine 705 (Y705), leading to dimerization and nuclear translocation. Once in the nucleus, these dimers bind to specific DNA-binding motifs in the promoter regions of target genes, initiating gene transcription. Notably, STAT3^Y705^ was readily detectable in TET3-containing protein complexes in MDMs (Figure 3C), supporting a physical interaction between TET3 and activated STAT3. To assess 5hmC modification at the *TGFB1* promoter, we performed hydroxymethylated DNA immunoprecipitation with a 5hmC-specific antibody coupled with qPCR (hMeDIP-qPCR) using our previous method (31). No difference in 5hmC modification was detected between the two groups (Figure 3D). Next, we performed methylated DNA immunoprecipitation using a 5mC-specific antibody coupled with qPCR (MeDIP-qPCR) following our previously established method (32). Results revealed reduced DNA methylation of the *TGFB1* promoter in Ad-TET3–infected versus Ad-GFP–infected MDMs (Figure 3E). These findings suggest that 5hmC modification occurs transiently prior to rapid demethylation. Indeed, the kinetics of the 5mC to 5hmC conversion and subsequent demethylation in vivo are highly context dependent (9). Collectively, our results indicate that TET3, recruited via activated STAT3, facilitates demethylation and chromatin remodeling, thereby enhancing *TGFB1* transcription in macrophages.

We further hypothesized that TET3 binds to a specific region of the *NLRP3* promoter via STAT3 interaction, thereby inducing 5hmC modification, chromatin remodeling, and active *NLRP3* transcription. To test this, we infected human MDMs with Ad-TET3 or Ad-GFP and performed ChIP-qPCR and hMeDIP-qPCR analyses. After screening multiple qPCR primer sets spanning the *NLRP3* promoter, we identified a region near the transcription start site that elicited TET3-dependent effects (Figure 3F). Compared with Ad-GFP–infected MDMs, TET3, STAT3, and H3K4me3 binding to this region was significantly increased in Ad-TET3–infected cells (Figure 3G). Similar increases in association were also observed in Ad-TET3–infected tumor cells (Supplemental Figure 4). hMeDIP-qPCR analysis with human MDMs revealed increased 5hmC modification in Ad-TET3 versus Ad-GFP conditions (Figure 3H). Together, these results suggest that TET3, via STAT3, is recruited to the *NLRP3* promoter, leading to 5hmC modification, an open chromatin conformation, and *NLRP3* transcription. Interestingly, neither the *TGFB1* promoter fragment (Figure 3A, –23 to +69) nor the *NLRP3* promoter fragment (Figure 3F, -42 to +35) contains a canonical STAT3-binding motif. However, this is not unexpected, as STAT3 has been shown to associate with promoter regions in the absence of consensus STAT3-binding sites (33).

Next, we identified a consensus STAT3-binding site within the human *IL1B* promoter (blue highlight, Figure 3I). We hypothesized that TET3 enhances *IL1B* expression by binding this promoter via STAT3, leading to chromatin remodeling and transcription activation. Results from TET3-oe MDMs (Figure 3, J–L) support this hypothesis.

A prior study reported that a STAT3-binding site present in the *CCL2* promoter mediates CCL2 transcription in human colorectal cancer cells (blue highlight, Figure 3M) (34). When TET3 was overexpressed in MDMs, we observed an increased association of TET3, STAT3, and H3K4me3 with this region (Figure 3N), consistent with TET3-dependent chromatin remodeling leading to transcription activation of *CCL2*. Using hMeDIP/MeDIP, we were unable to detect hydroxymethylation or methylation in this promoter region, suggesting a mechanism independent of DNA modification. This was not surprising, as TET proteins can function independently of catalytic activity (9). For example, TET2 binds to the mouse *Il6* promoter and recruits HDAC1/2 to suppress transcription without altering the DNA methylation status (35, 36). Similarly, TET3 inhibits IFN-β expression by recruiting HDAC1 to its promoter in a DNA modification–independent manner (21).

Our findings establish that TET3 promotes the expression of TGF-β1, NLRP3, IL-1β, and CCL2 in Toe-Macs via an epigenetic mechanism involving STAT3. The STAT3 SH2 domain is essential for Y705 phosphorylation, dimerization, and nuclear relocation. Stattic, a small molecule that specifically inhibits the STAT3 SH2 domain (37), significantly reduced TET3 binding to the 4 gene promoters, underscoring the critical role of activated STAT3 in docking TET3 to these regulatory regions (Supplemental Figure 5).

### Bobcat339 is a TET3-specific, molecular glue-like degrader.

**Figure 4:**
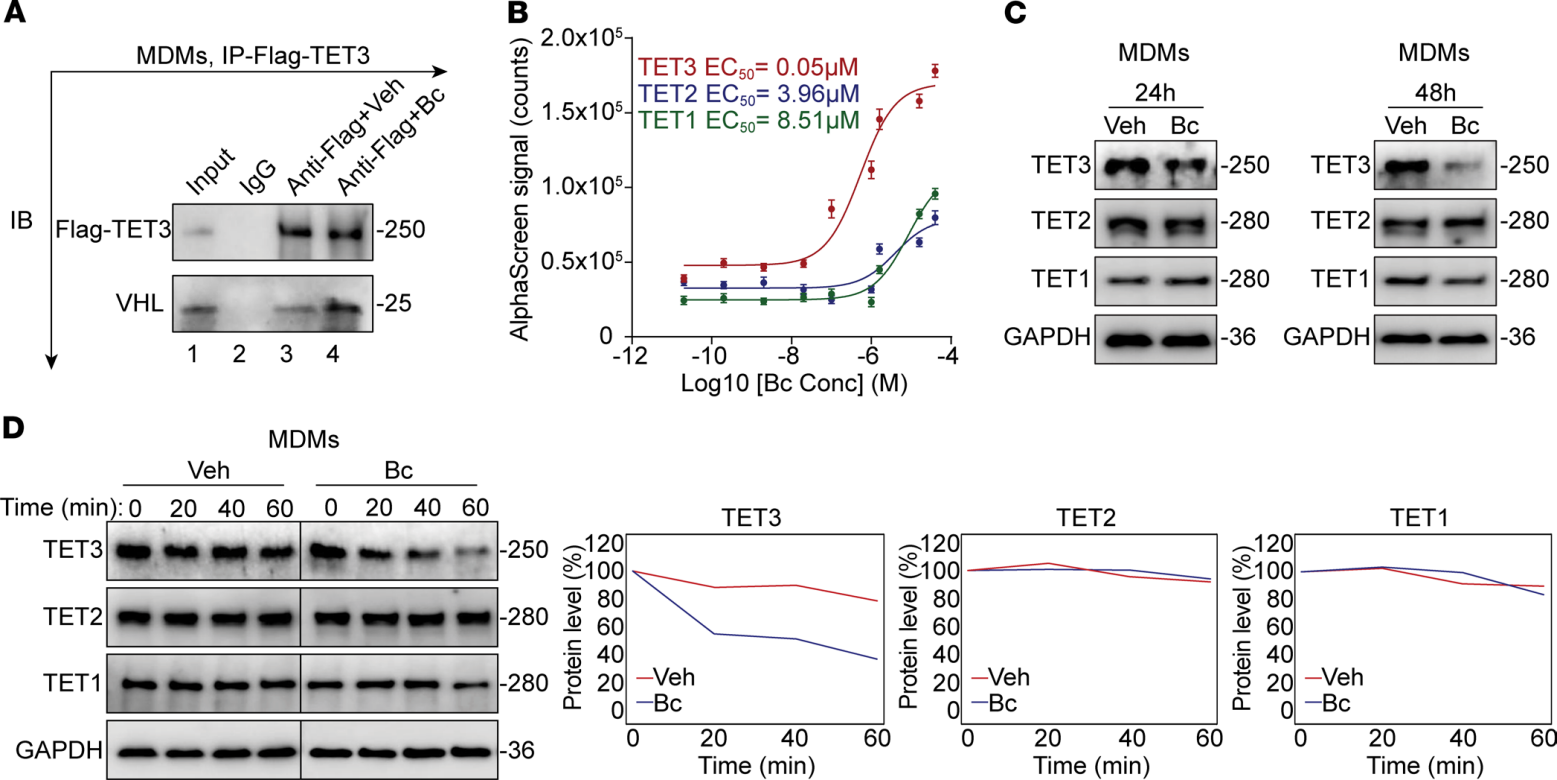
Bc destabilizes TET3 protein through enhancing a ternary complex formation with VHL. (**A**) Co-IP of Flag-TET3 and endogenous VHL in MDMs infected with Ad-TET3 (which expresses a Flag-tagged human TET3), with or without the presence of Bc, at 50 μM for 2 hours. (**B**) AlphaScreen of dose-response curves of Bc at different concentrations (conc) for binding of TET3, TET2, and TET1 to VHL. (**C**) Western blots of TET3, TET2, and TET1 in MDMs incubated for 24 hours or 48 hours with Veh or Bc at a final concentration of 10 μM. (**D**) MDMs were incubated for 2 hours with Veh or Bc at a final concentration of 10 μM, followed by time-course analysis of TET3, TET2, and TET1 in the presence of cycloheximide (CHX) at a final concentration of 50 μg/mL. Cells were harvested at 0, 20, 40, and 60 minutes after addition of CHX. Quantifications are displayed on the right. Western blot data are representative of 2–3 biological repeats.

Bobcat339 (Bc), a cytosine derivative with a molecular weight of 297.74, was rationally designed using in silico modeling to bind to the catalytic domains (CDs) of TET2 and TET1 to inhibit their catalytic activity (38). However, the binding affinities of Bc to TET proteins were not experimentally determined (38). It was later found that Bc alone was unable to inhibit TET enzymatic activity and that the observed effects were in fact mediated by contaminating Cu^2+^ (39). However, Bc appears to act as a molecular glue (MG). MGs are a unique class of small molecules that act to enhance weak, preexisting protein-protein interactions (40). MGs promote ternary complex formation by optimizing the existing interaction interface despite lacking a detectable affinity for at least 1 of the binding partners (40). Thus, detectable affinity toward either partner is not a prerequisite for MG function. The von Hippel Lindau (VHL) protein, the substrate recognition subunit of an E3 ubiquitin ligase, has been shown to target all 3 TET proteins for ubiquitination and subsequent proteasomal degradation (41). We found that Bc alone (without contaminating Cu^2+^) accelerates VHL-dependent degradation of TET3 in both human MDMs and mouse primary peritoneal macrophages (22). Using co-IP, we demonstrated that Bc enhanced the interaction between TET3 and VHL in human MDMs (Figure 4A). To confirm Bc-dependent ternary complex formation, we used AlphaScreen technology, a bead-based approach that relies on protein-protein interactions bringing donor and acceptor beads into proximity. When the beads are close (200 nm), excitation of donor beads leads to singlet oxygen release, which triggers light emission from the acceptor beads. The emitted light is directly proportional to the interaction strength. Biotinylated human VHL was immobilized to streptavidin-coated donor beads, while Flag-tagged CDs of human TET3, TET2, or TET1 were bound to anti-Flag antibody–coated acceptor beads. Donor and acceptor beads were combined in the presence or absence of increasing concentrations of Bc, and interaction signals were quantified. Sigmoid dose-response curves were obtained for TET3 (EC_50_ = 0.05 mM), TET2 (EC_50_ = 3.96 mM), and TET1 (EC_50_ = 8.51 mM), with Bc showing approximately 79- to 170-fold greater potency toward TET3 as compared with TET2 and TET1, respectively (Figure 4B). Consistently, treatment of human MDMs with Bc substantially reduced steady-state TET3 levels but not the levels of TET2 and TET1 (Figure 4C). Furthermore, Bc shortened the half-life of TET3 to 45 minutes without affecting TET2 or TET1 stability (Figure 4D). Collectively, these results support the conclusion that Bc acts as a TET3-specific degrader, likely via a molecular glue–like mechanism.

### Bobcat339 mimics the therapeutic effects of myeloid-specific Tet3 ablation in mouse models of MASH.

In endometriosis, signals from the DME (e.g., TGF-β1 and CCL2) drive TET3 overexpression (22), generating a unique Toe-Macs macrophage population that becomes dependent on elevated TET3 levels. Consequently, restoring TET3 to baseline levels in these cells induces apoptosis rather than reprogramming (22). As illustrated in Supplemental Figure 6A, homeostatic macrophages expressing a basal level of TET3 were not sensitive to Bc-induced degradation (compare lane 2 vs. lane 1). This aligns with the principle that transcripts with low expression are less susceptible to siRNA-mediated knockdown (42). Also, Bc treatment did not increase apoptosis in these basal-state cells (Supplemental Figure 6B). In contrast, Bc reduced TET3 levels (Supplemental Figure 6A, compare lane 4 vs. lane 3) and induced apoptosis (Supplemental Figure 6B) in TGF-β1–primed macrophages (Supplemental Figure 6B). These findings explain the high selectivity of Bc toward Toe-Macs and its lack of in vivo toxicity in endometriosis models (22).

We hypothesized that the Toe-Macs present in MASH livers are pathogenic and can be selectively eliminated due to their TET3 dependence. To test this, we used Bc and a *Tet3*-KO mouse model generated by crossing *Tet3^fl/fl^* mice with *LysM-Cre* mice to delete *Tet3* in the myeloid lineage (22). KO mice exhibited an approximately 90% reduction in *Tet3* expression without changes in *Tet2*. Myeloid lineage distribution, macrophage numbers, body weight, body composition, and fasting glucose remained unchanged (22), suggesting TET2/TET3 functional redundancy in monocyte development (22, 43). We hypothesized that KO mice would exhibit fewer Toe-Macs under MASH, while in WT mice, Toe-Macs induced by MASH would remain vulnerable to Bc-mediated depletion.

**Figure 5:**
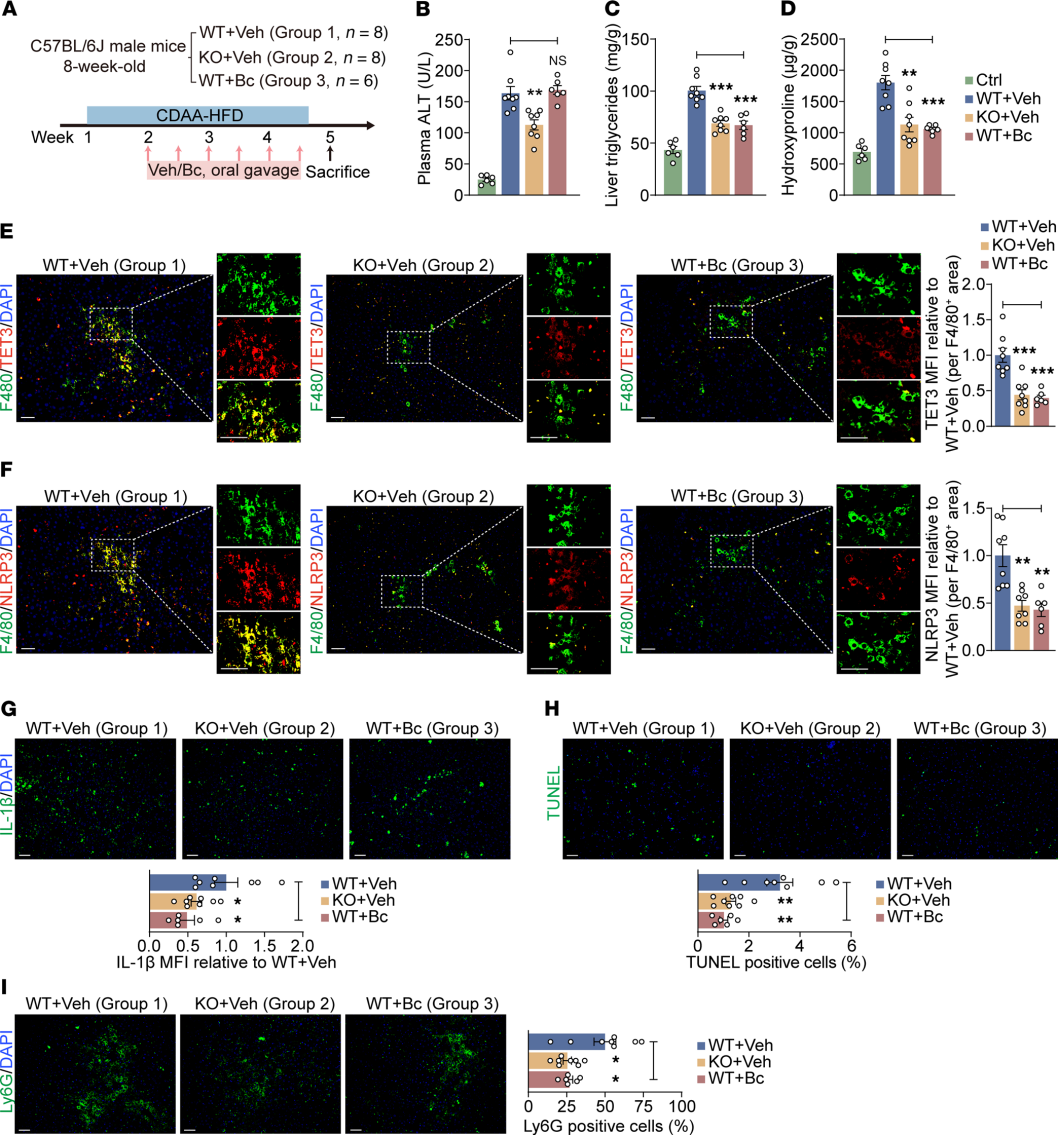
Toe-Macs are mechanistically linked to diet-induced MASH. (**A**) Schematic diagram of the experiments. (**B**–**D**) Plasma ALT (**B**), liver tissue triglycerides (**C**), and liver tissue hydroxyproline content (**D**) from mice treated as indicated. (**E**) Representative immunostaining for TET3 (red) in F4/80^+^ (green) macrophages with quantification of macrophage TET3 median fluorescence intensity (MFI) in liver tissue sections. (**F**) Immunostaining for NLRP3 (red) and F4/80 (green) in macrophages and quantification of macrophage NLRP3 MFI in liver tissue sections. (**G**) Immunostaining for IL-1β (green) and quantification of IL-1β MFI in liver tissue sections. (**H**) Photomicrographs and corresponding statistical analysis of TUNEL^+^ (green) cells. (**I**) Immunostaining for Ly6G (green) and quantification of Ly6G^+^ cells in liver tissue sections. All data represent the mean ± SEM. *n* = 6–8 mice per group. Each dot represents a mouse. *P < 0.05, **P < 0.01, and ***P < 0.001, by 1-way ANOVA with Tukey’s post test for the statistical differences versus the WT+Veh group. Scale bars: 25 μm.

First, we used a well-established choline-deficient, l–amino acid–defined, high-fat diet–induced (CDAA-HFD–induced) mouse MASH model (7, 12). WT and KO mice were fed the CDAA-HFD diet for 4 weeks, with vehicle (Veh) or Bc treatment administrated at 30 mg/kg twice weekly starting in week 2 (Figure 5A). WT mice fed normal chow were used as a control group (Ctrl). All mice were sacrificed at week 5. Compared with the Ctrl group, mice on the CDAA-HFD (groups 1–3) exhibited significantly elevated plasma ALT levels (Figure 5B), indicating liver damage. When group 2 (KO) and group 3 (Bc-treated) mice were compared with group 1 (WT+Veh), both showed significantly reduced liver triglycerides levels (Figure 5C) and hepatic hydroxyproline content (Figure 5D), indicating decreased steatosis and fibrosis. These reductions were further confirmed by H&E staining (Supplemental Figure 7A) and Picrosirius red/Fast Green staining (Supplemental Figure 7B). Additional analyses compared groups 1–3 to assess the therapeutic effects of TET3 ablation and Bc treatment.

Consistent with human snRNA-Seq and IHC data (Figure 1 and Supplemental Figure 2) and with mouse single-cell RNA-Seq (scRNA-Seq) findings (Supplemental Figure 8) (44), we found that Toe-Macs were readily detected in livers from group 1 mice (left panel, Figure 5E). The abundance of Toe-Macs was significantly reduced in group 2 (middle panel, Figure 5E) and group 3 (right panel, Figure 5E), suggesting that MASH induced Toe-Macs (group 1), but the induction was attenuated by either genetic ablation (group 2) or Bc treatment (group 3). Importantly, there was a concurrent reduction in NLRP3-oe Macs (Figure 5F), TGF-β1–oe Macs (Supplemental Figure 7C), CCL2-oe Macs (Supplemental Figure 7D), and IL-6–oe Macs (Supplemental Figure 7E) in mice of groups 2 and 3 as compared with group 1 mice. This supports in vivo regulation of the expression of the *NLRP3*, *TGFB1*, *CCL2*, and *IL6* genes by TET3. These findings were further supported by bulk liver tissue protein analysis, which showed a strong decrease in TET3, NLRP3, and TGF-β1 expression in groups 2 and 3 relative to group 1 (Supplemental Figure 7F). The same dataset showed reduced levels of α–smooth muscle actin (α-SMA) and collagen 1 (COL1A) in groups 2 and 3 (Supplemental Figure 7F). α-SMA marks hepatic stellate cell (HSC) activation, a key step in fibrogenesis. TGF-β1 potently stimulates HSC activation. Activated HSCs transdifferentiate into myofibroblasts, which secrete extracellular matrix (ECM) proteins such as COL1A (45).

Compared with group 1, IL-1β expression was lower in the livers of group 2 and group 3 mice (Figure 5G), consistent with in vitro findings that TET3 stimulated macrophage IL-1β production (Figure 2). TUNEL assays revealed fewer apoptotic cells in liver tissue from group 2 and group 3 mice (Figure 5H), suggesting reduced hepatocellular death. Neutrophil infiltration was also significantly lower in these groups, consistent with decreased hepatic inflammation (Figure 5I) (12).

We observed similar results in a second mouse model of MASH (Supplemental Figures 9 and 10). We conclude that Toe-Macs are pathogenic and can be selectively eliminated through genetic ablation or pharmacological intervention using Bc. The pathogenicity is likely driven, at least in part, by TET3-induced upregulation of NLRP3, IL-1β, IL-6, CCL2, and TGF-β1, all key mediators of the inflammatory and fibrotic DME.

### Toe-Macs are induced by the TME of NSCLC.

**Figure 6:**
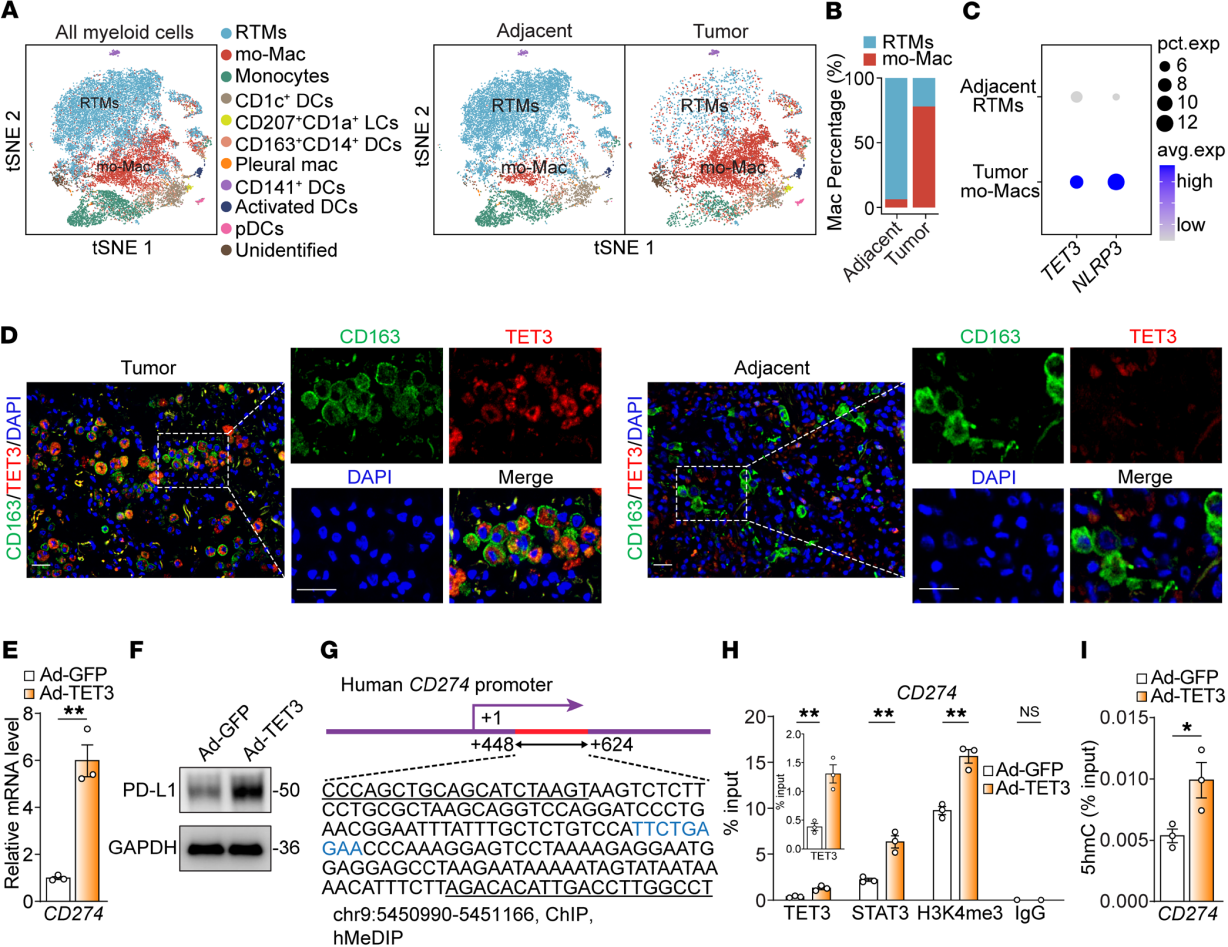
Toe-Macs present in human NSCLC epigenetically promote PD-L1 expression. (**A**) Dimension reduction plots showing all myeloid cells from adjacent and tumor lung samples. pDCs, plasmacytoid DCs. (**B**) Bar plot showing macrophage percentages of RTMs and mo-Macs in adjacent and tumor tissue. (**C**) Dot plot showing expression *TET3* and *NLRP3* in adjacent RTMs and tumor mo-Macs. (**D**) Immunostaining for TET3 (red) and CD163 (green) in human NSCLC tumor tissue and adjacent normal tissue. Scale bars: Scale bar: 50 μm. (**E** and **F**) CD274/PD-L1 expression in MDMs infected with Ad-GFP or Ad-TET3, assessed by qRT-PCR (**E**, RNA harvested at 12 hours) and Western blotting (**F**, protein harvested at 24 hours). (**G**) PCR-amplified fragment of the *CD274* promoter is marked in red, with the zoomed-in sequence shown underneath. The PCR primer sequences are underlined with the STAT3-binding site colored blue. (**H**) MDMs were treated as in Figure 3B. ChIP-qPCR was performed to detect enrichment of the specific region in the *CD274* promoter outlined in **G**. (**I**) MDMs were treated as in Figure 3B, followed by hMeDIP-qPCR to detect 5hmC levels in the specific region in the *CD274* promoter outlined in **G**. All data represent the mean ± SEM. *P < 0.05 and **P < 0.01, by 2-tailed Student’s *t* test. Western blot data are representative of 2 biological repeats. Refer to Supplemental Table 1 for detailed patient sample information.

The lung contains 2 distinct populations of RTMs, alveolar macrophages (AMs) and interstitial macrophages (IMs), both of which are essential for maintaining lung homeostasis and host defense (46). These macrophages originate from the yolk sac or fetal liver and are gradually replenished by mo-Macs with age (46). Analysis of a publicly available scRNA-Seq dataset from a cohort of patients with NSCLC (47) revealed mo-Macs that were predominantly associated with tumor tissue compared with adjacent normal tissue (Figure 6, A and B). This finding aligns with the notion that, while both AMs and IMs can contribute to TAMs, mo-Macs dominate the TME during tumor growth (48–50). Compared with RTMs in the normal tissue, a subset of mo-Macs in the tumor overexpressed TET3 and NLRP3 (Figure 6C), consistent with mouse scRNA-Seq studies (Supplemental Figure 11) (51). Further inspection of the human scRNA-Seq dataset (47) identified a population of tumor cells expressing high levels of TGF-β1 and CCL2 (Supplemental Figure 12). Since macrophage TET3 expression can be upregulated by TGF-β1 and CCL2 (Figure 2A and Supplemental Figure 3A), we propose that Toe-Macs are induced by the TME. To validate this, we performed IHC analysis on tissue samples from patients with NSCLC using CD163 as a macrophage marker, given that most human lung macrophages express CD163 (46, 47). Consistent with the scRNA-Seq data, we detected Toe-Macs in tumor tissues but not in adjacent normal tissue (Figure 6D and Supplemental Figure 13).

As a major driver of epithelial-mesenchymal transition (EMT), TGF-β1 plays a key role in NSCLC development, immune evasion, and metastasis. TAMs contribute to tumor-associated inflammation and immunosuppression via multiple mechanisms. TAM-derived IL-1β, TGF-β1, CCL2, and IL-6 can (a) inhibit cytotoxic T cell infiltration into the tumor stroma, (b) suppress the cytotoxic function of T cells and NK cells, (c) induce cytotoxic T cell exhaustion, (d) promote the transformation of normal fibroblasts into tumor-promoting, cancer-associated fibroblasts (CAFs), and (e) recruit myeloid-derived suppressor cells (MDSCs) and immune-suppressive Tregs to the TME. Additionally, TAM-derived PD-L1 (a ligand for the immune checkpoint protein PD-1) contributes to cytotoxic T cell dysfunction (13, 14).

We hypothesized that the Toe-Macs in NSCLC are pathogenic, in part due to enhanced expression of the key tumor-promoting factors NLRP3, IL-1β, TGF-β1, CCL2, IL-6, and PD-L1 (encoded by *CD274*). Mining the human scRNA-Seq data (47) revealed a positive correlation between TET3 expression and each of these 6 genes (Supplemental Figure 14). In human MDMs, TET3 epigenetically upregulates NLRP3, IL-1β, TGF-β1, and CCL2 (Figure 3) as well as enhances IL-6 production by suppressing let-7 expression (22). Together, these findings support a regulatory role for TET3 in driving the expression of NLRP3, IL-1β, TGF-β1, CCL2, and IL-6 in Toe-Macs in NSCLC. To determine how TET3 regulates PD-L1, we induced TET3 overexpression in human MDMs, which resulted in increased CD274 expression (Figure 6, E and F). ChIP-qPCR analysis revealed enhanced binding of TET3, STAT3, and H3K4me3 at *CD274* containing a consensus STAT3-binding site (blue highlight, Figure 6G) in TET3-oe cells (Figure 6H). This was accompanied by increased 5hmC modification at the same promoter region (Figure 6I). This STAT3 binding site has previously been implicated in regulating *CD274* transcription in human NSCLC cells (52). Importantly, STAT3-dependent recruitment of TET3 was confirmed by decreased TET3 binding to the *CD274* promoter in cells treated with Stattic (Supplemental Figure 5). Collectively, these data support an epigenetic mechanism through which TET3, in concert with STAT3, activates the expression of NLRP3, IL-1β, TGF-β1, CCL2, and PD-L1 and enhances IL-6 posttranscriptionally. These TET3-driven programs in TAMs likely contribute to immunosuppression and tumor progression in NSCLC.

### Bobcat339 recapitulates the therapeutic effects of myeloid-specific Tet3 ablation in a mouse model of NSCLC.

**Figure 7:**
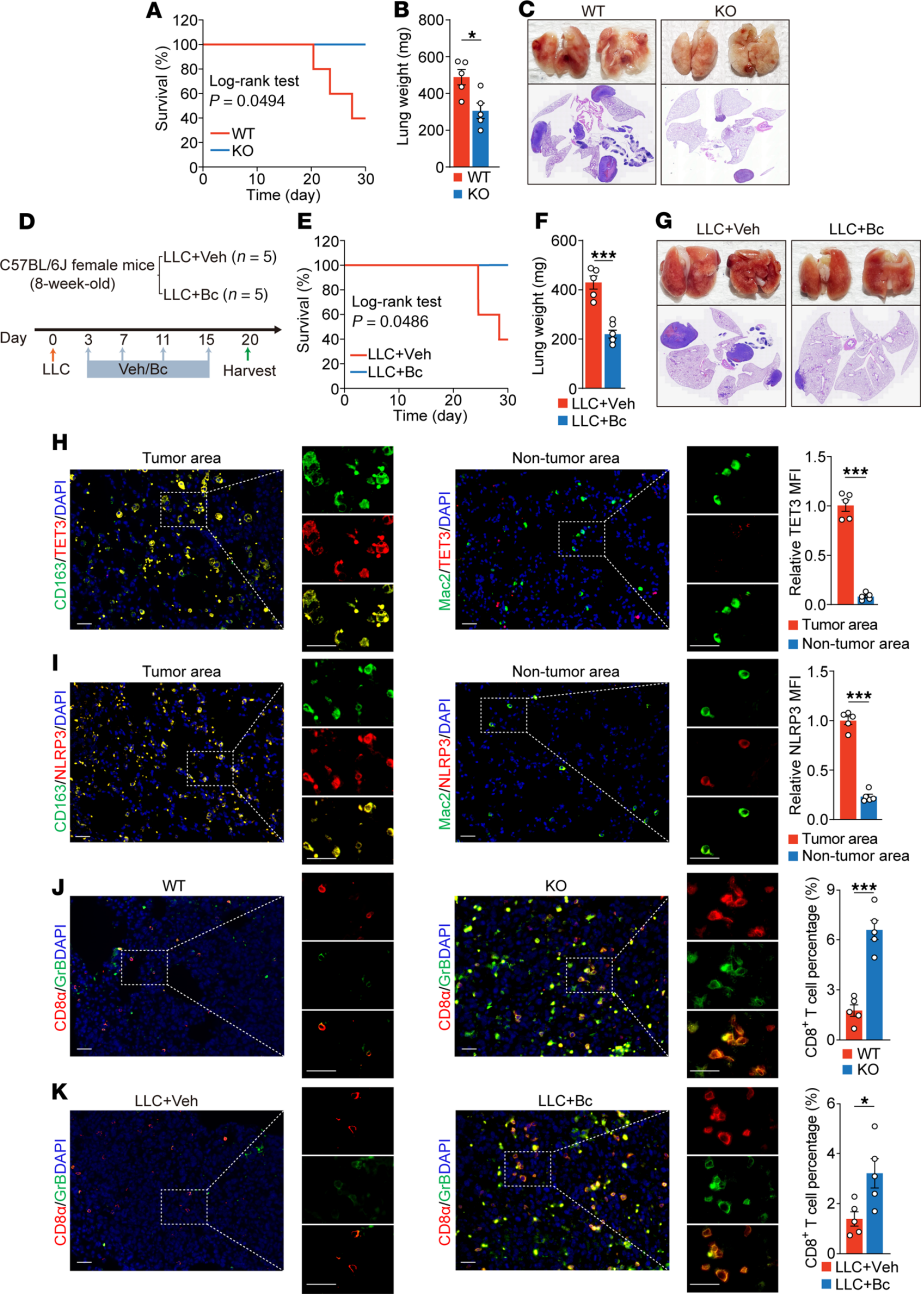
Toe-Macs contribute to an immunosuppressive TME in NSCLC. (**A**) Survival rates of WT and KO mice after LLC inoculation. (**B**) Lung weights of WT and KO mice injected with LLC cells. (**C**) Macroscopic images and H&E stains (original magnification, ×10) of lungs from WT and KO mice injected with LLC cells. (**D**) Experimental design. (**E** and **F**) Survival rates and lung weights of mice treated as in **D**. (**G**) Macroscopic images and H&E stains (original magnification, ×10) of lungs from mice treated as in **D.** (**H**) Immunostaining for TET3 (red) in CD163^+^ macrophages (green) in tumor areas and TET3 (red) in Mac2^+^ macrophages (green) in nontumor areas, and quantification of macrophage TET3 MFI in tumor and nontumor areas. (**I**) Immunostaining for NLRP3 (red) in CD163^+^ macrophages (green) in tumor areas and NLRP3 (red) in Mac2^+^ macrophages (green) in nontumor areas, and quantification of macrophage NLRP3 MFI in tumor and nontumor areas. (**J**) Costaining for CD8a (red) and GrB (green) in tumor areas in WT and KO mice, and quantification of CD8^+^ T cell percentages in each group. (**K**) Costaining for CD8a (red) and GrB (green) in tumor areas of Veh- and Bc-treated mice, and quantification of CD8^+^ T cell percentages in each group. All data represent the mean ± SEM. *n* = 5 per group. *P < 0.05 and ***P < 0.001, by 2-tailed Student’s *t* test. Scale bars: 50 μm.

We hypothesized that eliminating Toe-Macs would confer therapeutic effects. To test this, we used a syngeneic lung cancer mouse model in combination with either myeloid-specific *Tet3* KO (KO mouse model) or Bc. First, WT and KO female mice received tail vein injections of Lewis lung carcinoma (LLC) cells to establish metastatic lung cancer. The KO mice had significantly increased survival compared with WT animals (Figure 7A). Additionally, KO mice had significantly lower lung weights (Figure 7B) and fewer LLCs (Figure 7C), indicating reduced tumor burden. These data demonstrate that myeloid-specific *Tet3* ablation attenuated lung cancer progression.

Next, we established metastatic LLC in WT female mice and administrated Bc or Veh at 3 mg/kg every 4days, starting 3 days after tumor inoculation (Figure 7D). The Bc-treated mice showed significantly prolonged survival (Figure 7E), lower lung weights (Figure 7F), and fewer lung metastases (Figure 7G), demonstrating that Bc effectively mitigated tumor progression.

In mice, CD163 is not expressed by lung macrophages at homeostasis but is upregulated in the TME (46). In both genotypes (WT and KO) and treatment groups (Veh and Bc), CD163^+^ Toe-Macs were detected within tumor regions (Figure 7H) expressing high levels of NLRP3 (Figure 7I), IL-1β (Supplemental Figure 15A), TGF-β1 (Supplemental Figure 15B), CCL2 (Supplemental Figure 15C), IL-6 (Supplemental Figure 15D), and PD-L1 (Supplemental Figure 15E). In contrast, these CD163^+^ Toe-Macs were absent in nontumor regions, although macrophages lacking TET3 expression were detectable using a Mac2-specific antibody (53) (Figure 7H). This finding mirrors human NSCLC, in which Toe-Macs were localized to the tumor but not adjacent normal tissue (Figure 6D and Supplemental Figure 13). Moreover, macrophages in nontumor areas exhibited minimal expression of NLRP3 (Figure 7I), IL-1β (Supplemental Figure 15A), TGF-β1 (Supplemental Figure 15B), CCL2 (Supplemental Figure 15C), IL-6 (Supplemental Figure 15D) and PD-L1 (Supplemental Figure 15E). An immunosuppressive TME is marked by impaired cytotoxic T lymphocyte (CTL) activity. A robust antitumor response is characterized by infiltration of CD8^+^ CTLs expressing granzyme B (GrB), a key cytotoxic protease. CTLs function as “serial killers,” eliminating multiple tumor cells by delivering cytotoxic granules containing GrB, thereby inducing apoptosis (54). Compared with WT mice, tumors from both KO and Bc-treated mice showed a significant increase in CD8^+^ T cell infiltration (Figure 7, J and K). Moreover, CD8^+^ T cells from WT and Veh-treated mice had lower GrB expression levels. These findings are consistent with studies of human NSCLC showing that soluble tumor-derived factors suppress GrB expression in infiltrating CD8^+^ T cells (55), with TGF-β1 specifically shown to repress GrB transcription and cytotoxic function (56). We conclude that Toe-Macs contributed to the establishment and maintenance of an immunosuppressive TME by promoting the expression of NLRP3, IL-1β, TGF-β1, CCL2, IL-6, and PD-L1. Targeting these macrophages either genetically or pharmacologically via Bc enhanced CTL infiltration and function, offering a novel therapeutic strategy for NSCLC.

### Endometriosis macrophages share the hallmark of Toe-Macs identified in MASH and NSCLC.

Our previous studies identified Toe-Macs within endometriotic lesions (22). These cells exhibit disease-promoting properties by producing high levels of IL-1β and IL-6 through a posttranscriptional regulatory mechanism involving the *let-7* microRNA family (22). Depletion of these macrophages either via myeloid-specific *Tet3* ablation or treatment with Bc markedly reduced endometriotic lesion burden in mice (22). On the basis of these findings, we hypothesized that Toe-Macs in endometriosis share functional similarity with their counterparts in MASH and NSCLC. Supporting this hypothesis, analysis of human scRNA-Seq data (57) revealed a predominant enrichment of both Toe-Macs and NLRP3-oe Macs in ectopic endometriotic lesions compared with paired eutopic endometrial tissue (Supplemental Figure 16). To extend these findings, we performed IHC on archived mouse tissue from prior studies (22). Toe-Macs and NLRP3-oe Macs were abundantly detected in endometriotic lesions from WT mice but were significantly reduced in lesions from KO or Bc-treated mice (Supplemental Figure 17, A and B). Similar reductions were observed for IL-1β–oe Macs, TGF-β1–oe Macs, CCL2-oe Macs, and IL-6–oe Macs (Supplemental Figure 17, C–F). These results collectively support the notion that Toe-Macs present a common, pathologic macrophage phenotype that contributes to disease progression across inflammatory and fibrotic conditions including endometriosis, MASH, and NSCLC.

## Discussion

**Figure 8:**
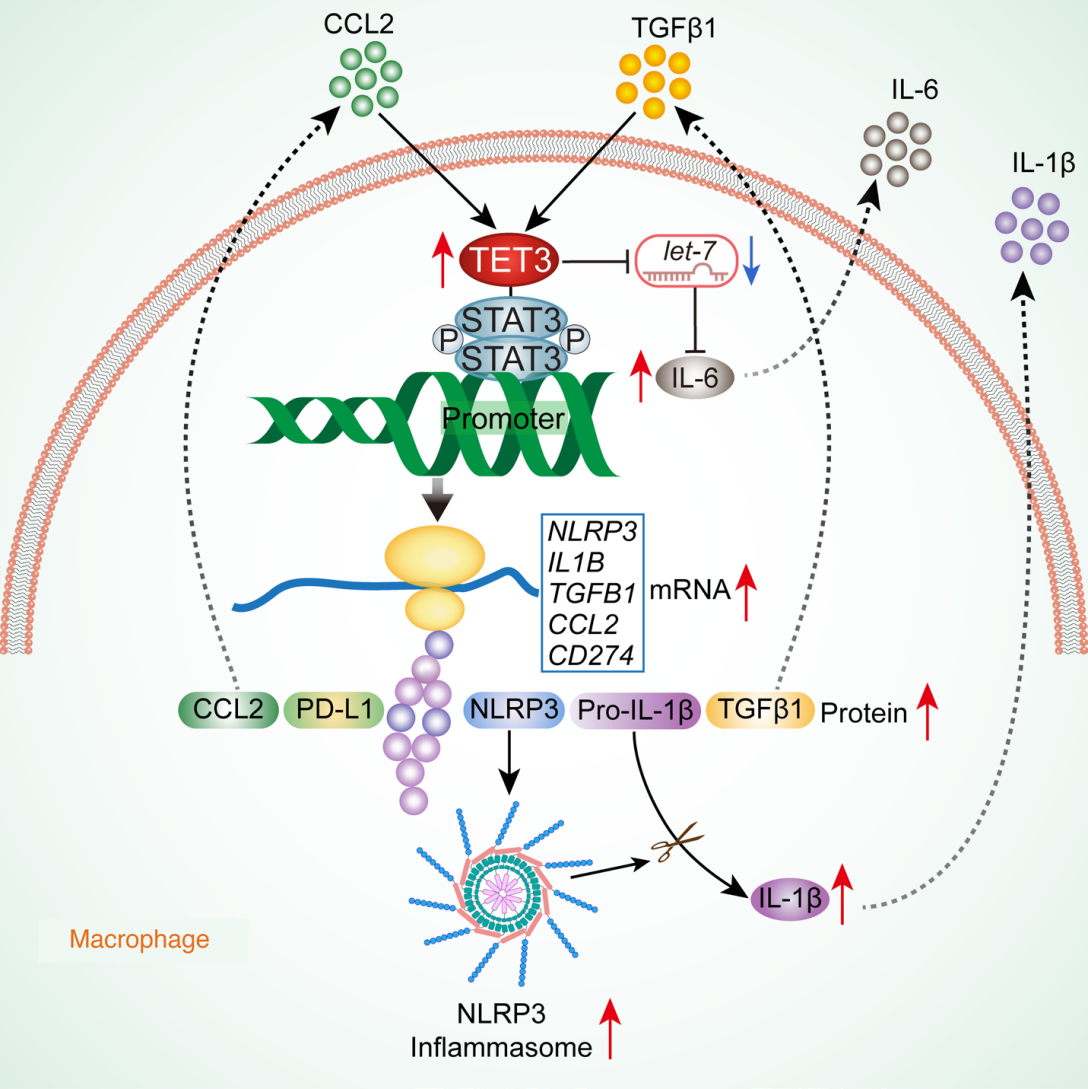
Proposed model. TET3 overexpression is induced by CCL2 and TGF-β1, which are commonly present in the DME. TET3 downregulates *let-7* expression, leading to increased production of IL-6. TET3 is recruited to specific gene promoters through interaction with activated STAT3. By binding to the promoters of *NLRP3*, *IL1B*, *TGFB1*, and *CD274*, TET3 induces 5hmC DNA modification and demethylation and creates an open chromatin state facilitating transcription. In the case of *CCL2*, a mechanism independent of DNA modification is involved. This results in an increase in protein production of NLRP3, pro–IL-1β, TGF-β1, CCL2, and PD-L1, as well as enhanced NLRP3 inflammasome activity, which in turn promotes the maturation and release of IL-1β. Furthermore, TGF-β1 and CCL2 released from the Toe-Macs can act as autocrine factors to increase TET3 expression, creating a positive feedback loop with TET3.

Combining sc/snRNA-Seq data analyses, primary macrophage studies, genetic KO mouse models, and pharmacological interventions, we report the identification of pathogenic DAMs, Toe-Macs, in major human diseases associated with chronic inflammation. Despite their heterogeneity, these macrophages share the hallmark of TET3 overexpression–induced NLRP3 hyperactivation leading to IL-1β overproduction. IL-1β is a major cytokine responsible for the establishment and maintenance of a chronic inflammatory/immunosuppressive state in many human diseases. Importantly, TET3 also directly activates IL-1β transcription at the epigenetic level, potentially providing synergistic effects in promoting inflammation. As illustrated in our model (Figure 8), TET3 overexpression in these macrophages is induced by common factors present in the DME. TET3 downregulates *let-7* expression, leading to increased production of IL-6 (22). Restoring let-7 levels may suppress Toe-Mac function and/or IL-6 output, but this may not be a practical therapeutic strategy. The reason is that let-7 has numerous targets, and increasing its levels would likely cause significant off-target effects. TET3 is recruited to specific gene promoters through interaction with STAT3 known to bind to DNA in a sequence-specific manner. By binding to the promoters of *NLRP3*, *IL1B*, *TGFB1*, and *CD274*, TET3 induces 5hmC DNA modification and demethylation and creates an open chromatin state (marked by H3K4Me3) facilitating transcription. In the case of *CCL2*, a mechanism independent of DNA modification appears to be involved. This results in increased protein production of NLRP3, pro–IL-1β, TGF-β1, CCL2, and PD-L1, as well as enhanced NLRP3 inflammasome activity, which in turn promotes the maturation and release of IL-1β. Furthermore, TGF-β1 and CCL2 released from Toe-Macs may act as autocrine factors to increase TET3 expression, creating a positive feedback loop with TET3. TGF-β1 and CCL2 significantly increase TET3 expression at both the mRNA and protein levels (22) (Figure 2A). Their combined effect on TET3 upregulation appears synergistic (Figure 2B), adding a crucial regulatory dimension to the Toe-Mac–mediated disease mechanism. However, the precise way this synergistic regulation occurs requires further investigation.

TET3 regulates the expression of NLRP3, IL-1β, CCL2, and PD-L1 at the epigenetic level was not previously documented, nor was the mechanistic involvement of STAT3 in TET3-mediated regulation of TGF-β1 previously reported. It was initially unexpected that TET3 would use the same adaptor protein, STAT3, to regulate all 5 genes, *NLRP3*, *IL1B*, *TGFB1*, *CCL2*, and *CD274*. However, given their key roles in promoting inflammation and immune suppression, such coordinated regulation might be highly effective and advantageous to pathogenic macrophages. The precise mechanism by which STAT3 recruits TET3 to its 5 target genes remains an area requiring further elucidation. A fundamental question pertains to the nature of the TET3-STAT3 complex itself: Is their interaction direct, or is it mediated by one or more bridging proteins? Furthermore, it is not yet understood whether the composition of the TET3-STAT3–containing complexes is consistent across all 5 genes, or if distinct components are involved depending on the specific gene being regulated. Our discovery provides a mechanistic understanding of why Toe-Macs from such strikingly different disease types share pathogenic properties. Future studies such as genome-wide DNA hydroxymethylation/methylation profiling and elucidation of enzymatic versus nonenzymatic functions of TET3 will be important.

Macrophages are highly plastic; they change surface and intracellular markers and functional properties in a disease/disease stage–dependent manner (1). Also, many populations of myeloid cells express overlapping surface and intracellular markers. Thus, the use of flow cytometry to identify specific populations of DAMs has inherent limitations in that there exist no true macrophage-specific markers that can be used either singly or in combination in flow cytometry to accurately identify all macrophages that are pathogenic or protective. Furthermore, information about anatomical localization is lost during flow cytometry sample preparation. We believe that our combinatorial approaches (sc/snRNA-Seq, IHC, in vitro and in vivo, genetic and pharmacological manipulations) have proven to be suitable to identify at least a large fraction of pathogenic DAMs. While it is yet to be determined which subpopulations of macrophages undergo TET3 overexpression during disease, the myeloid-specific *Tet3*-KO or Bc treatment is anticipated to eliminate the majority of Toe-Macs irrespective of their developmental origins, other molecular features, and surface and intracellular markers.

Our results are in apparent contrast with several elegant studies indicating that macrophage TET2 functions in a divergent manner to restrain the expression of proinflammatory cytokines and chemokines (4, 7, 35, 36, 43, 53, 58–60). In these studies, a positive correlation between TET2 deficiency and exaggerated inflammatory responses (i.e., increased production of IL-1β and IL-6) was observed, but the exact mechanism of action was unresolved in most of the studies. It was not determined which genes serve as critical direct targets of TET2, except in 2 studies (35, 36) demonstrating that TET2 selectively binds to the *Il6* promoter through interaction with the transcription factor IκBζ and recruits HDAC1/-2 to suppress transcription. Notably, TET3 expression appeared to be increased in at least 1 of the studies (7). Furthermore, TET3 was reported to inhibit expression of the type I IFN IFN-β by recruiting a repressive complex containing HDAC1 to the promoter of *Ifnb1* (21). At this time, we cannot rule out the possibility that both TET2 and TET3 contribute to hyperinflammatory responses, but in most cases with opposing effects and perhaps via different mechanisms. However, our data clearly demonstrate that TET3 is an important regulator of inflammation and acts through activation of multiple proinflammatory genes.

TET2 and TET3, but not TET1, proteins are broadly detected in adult human tissues (Supplemental Figure 18A) (61). Interestingly, TET2 and TET3 often exhibit functional redundancy. For instance, mice with skeletal muscle–specific Tet3 ablation show normal development and metabolism and are even resistant to diet-induced insulin resistance (62). Similarly, we and others have found that deleting either Tet2 or Tet3 individually in myeloid cells does not significantly alter the steady-state distribution or numbers of monocytes and macrophages (22, 35, 43). This suggests that, often, simultaneous ablation of both Tet2 and Tet3 is necessary to observe noticeable defects (9).

Given the functional redundancy of TET proteins, it is unlikely that TET3 knockdown in macrophages would significantly alter immune responses. First, type I IFNs (IFN-β and IFN-α) are crucial for host defense against viral infections (63). TET3 in macrophages actually suppresses type I IFN production (21), so reducing TET3 is not expected to dampen the host’s ability to fight viral infections. Second, macrophage TET2 expression is essential for antibacterial responses, protecting against conditions like peritonitis and abdominal sepsis, partly by inhibiting proinflammatory cytokines such as IL-6. (59). Since TET3 knockdown in macrophages does not affect TET2 expression (22), macrophage TET3 knockdown will probably not negatively affect the host’s defense against infections. Future studies should include comprehensive immunophenotyping of nonmacrophage cells to fully rule out broader immune effects.

Importantly, our findings suggest that Bc does not induce apoptosis in healthy macrophages but rather does so in disease-induced macrophages (Supplemental Figure 6). This suggests that DME factors not only increase TET3 but also alter the expression of other genes, making macrophages dependent on TET3 overexpression for survival. Future studies comparing global gene expression profiles in DME factor–primed macrophages, with and without TET3 knockdown, will help identify the gene pathways that control cell viability in these contexts. Moreover, the fact that macrophages not overexpressing TET3 were plentiful in KO and Bc-treated mice (Figure 5E, Supplemental Figure 10A, and Supplemental Figure 17A) indicates that TET3-targeted therapy does not have a major effect on macrophages involved in resolving disease or promoting tissue regeneration. Thus, targeting Toe-Macs while leaving disease-resolving macrophages intact has significant translational importance.

Curiously, we observed a positive correlation between TET3 expression and that of VHL in macrophages from MASH, NSCLC, and endometriosis (Supplemental Figure 19A), suggesting that TET3 may regulate VHL expression. Indeed, we detected increased VHL expression at both the mRNA and protein levels in TET3-oe human MDMs (Supplemental Figure 19B) and mouse BMDMs (Supplemental Figure 19C). We believe this finding is significant because Bc promotes TET3 degradation in a VHL-dependent manner (22). This mechanism is particularly relevant to the anticancer drug ABT263, an inhibitor of BCL-X_L_, has shown promise but was limited by dose-dependent platelet toxicity. Converting ABT263 into a proteolysis-targeting chimera (PROTAC) that directs BC_L_-XL to VHL-mediated degradation helped to overcome this adverse effect. This improvement was attributed to increased expression of both BCL-X_L_ and VHL in tumor cells but not in platelets (64). Thus, TET3-mediated upregulation of VHL may further enhance the specificity of Bc action, potentially providing a dual cell-specific targeting mechanism.

The favorable safety profile of Bc, a TET3-specific degrader, is noteworthy. Intravenous or oral administration of Bc showed high bioavailability (Supplemental Figure 18B) and no discernible side effects in mice. Female mice injected intraperitoneally with Bc exhibited no liver toxicity, body weight changes, or adverse effects on fertility (22, 65). Similarly, male mice treated orally with Bc showed no liver toxicity (Supplemental Figure 18D) or changes in myeloid development and distribution (Supplemental Figure 18E).

This safety, combined with the specificity of Bc as a TET3 (but not TET2 or TET1) degrader, its VHL-dependent mechanism of TET3 degradation, its selectivity toward disease-induced Toe-Macs over homeostatic ones, its attractive absorption, distribution, metabolism, and excretion (ADME) characteristics (Supplemental Figure 18C), the lack of significant binding to 44 safety screen targets and 97 kinases (data not shown), and the broad functional redundancy of TET2 and TET3, all contribute to why mice with myeloid-specific *Tet3* ablation or those treated with Bc showed no apparent side effects. These promising safety profiles and lack of off-target effects suggest that Bc could be a potential therapeutic agent for various diseases, operating through the same mechanism. For instance, in treating MASH, a significant unmet clinical need, Bc could serve as a powerful antiinflammatory and antifibrotic agent in combination therapy, complementing existing treatments like resmetirom (10).

## Methods

Detailed information can be found in the Supplemental Methods.

### Sex as a biological variable.

Male and female mice and samples from both male and female participants were used in our studies.

### Statistics.

The statistical analyses for each figure are indicated in the legends. All statistical analyses were performed using GraphPad Prism 10 for Windows (GraphPad Software) and are presented as the mean ± SEM. Two-tailed Student’s *t* tests (unless otherwise indicated) were used to compare means between groups. A *P* value of less than 0.05 was considered significant.

### Study approval.

The present studies in mice and studies involving the use of human blood samples were reviewed and approved by the Yale University Institutional Animal Care and Use Committee and the Yale University Human Investigation Committee.

### Data availability.

All study data are included in the article, the supplemental materials, and the Supporting Data Values file.

## Author contributions

BL, Y Dai, ZW, and Y Du performed experiments, analyzed data, and contributed to manuscript preparation. JS performed experiments using the WD-induced MASH model. HL performed human NSCLC IHC experiments. SB performed flow cytometric analysis. AK provided intellectual insights into Bobcat339. YHY provided important insights into cancer biology. XZ provided expertise on liver and lung pathology and performed data analyses and interpretation. MV and YS provided insights onto molecular glues and AlphaScreen analysis. HST contributed reagents and provided important intellectual insights into endometriosis. PW provided critical discussion on immunology and NLRP3. GGC contributed critical discussions throughout the work. YH and DL designed and supervised the study and wrote the manuscript. SZ contributed insights into endometriosis biology.

## Funding support

Yale Discover to Cure Program (to YH).
Paul Titus Scholar Award (to YH).
Albert McKern Fund (to YH).
Blavatnik Family Foundation (to YH).
National Natural Science Foundation of China (grant no. 82371647, to DL).

## Supplementary Material

Supplemental data

Unedited blot and gel images

Supplemental table 1

Supporting data values

## Version 1 08/12/2025

In-Press Preview

## Version 2 11/03/2025

Electronic publication
