# Supplementary material for: TET3 is a common epigenetic immunomodulator of pathogenic macrophages
Source: J Clin Invest. 2025 Aug 12;135(21):e194879. doi: 10.1172/JCI194879 (PMC12578387; doi:10.1172/JCI194879)
Supplement: Supplemental data [file jci-135-194879-s294.pdf]

Supplemental Information

TET3 is a common epigenetic immunomodulator of pathogenic macrophages

Files:

Supplemental Methods

Supplementary Figures 1-19

Supplemental Table 2

Supplemental Methods

Human samples
The use of human blood samples was approved by the Yale University Human Investigation Committee. Human peripheral blood mononuclear cells (PBMCs) were isolated and enriched using density gradient centrifugation. Macrophage differentiation was induced by treating PBMCs with recombinant human macrophage colony-stimulating factor (M-CSF, Gibco, PHC9504). Briefly, blood samples were collected from voluntary donors into sterile EDTA (K2) tubes (BD #366643) and diluted 1:1 in phosphate-buffered saline (PBS). An equal volume of the diluted blood was carefully layered onto Ficoll-Paque PLUS density gradient medium (Cytiva, 17144003) at a 1:1 ratio and centrifuged at 400 × g for 20 minutes at room temperature (RT) with no acceleration or brake. The PBMC layer was harvested and washed twice with PBS by centrifugation at 1200 rpm for 8 minutes at 4°C. To induce macrophage differentiation, PBMCs were resuspended in growth medium (RPMI 1640 supplemented with 10% fetal bovine serum [FBS, Gibco, 16140-017], 1% Anti-Anti [Gibco, 15240-062], and 50 ng/mL M-CSF). Cells were seeded into 24-well plates at a density of 2.5×10^5 cells per well or into 6 cm culture dishes at 2×10^6 cells per dish and maintained at 37°C in a 5% CO_2 humidified incubator. Nonadherent cells were removed after 7 days, and media were refreshed every 3 days.

Formalin-fixed, paraffin-embedded (FFPE) human tissue blocks were obtained from the Yale Pathology Tissue Service. The samples included normal livers (n=4), MASH livers (fibrosis stage 0-3, n=4 patients), and lung adenocarcinoma with matched normal adjacent tissues (n=3 patients). Refer to Supplemental Table 1 for detailed patient sample information.

Single-nucleus/single-cell RNA sequencing data analyses
Previously published, integrated sn/scRNA-seq datasets were retrieved from the Gene Expression Omnibus (GEO) database or other indicated repositories for re-analysis using Seurat v5 package in R studio Server (R version 4.3.2 (2023-10-31) - "Eye Holes"). GEO accession numbers or links are listed below. Layer integration was performed to integrate layers of different patients before running the Seurat functions of RunTSNE, RunUMAP and FindNeighbors. Gene expression levels were compared using Seurat DotPlot function.

Human liver snRNA-seq data (related to Figure 1 and Supplemental Figure 19) were previously integrated by Carlessi et al, and the data can be found at https://doi.org/10.17632/w7yh4yjvbw.2. Human NSCLC scRNA-seq data (related to Fig. 6 and Supplemental Figures 14, 12, and 19) were retrieved from GSE131907. Human endometriosis scRNA-seq data (related to Figure 8 and Supplemental Figures 16 and 19) was retrieved from GSE17964. Mouse liver snRNA-seq data (Supplemental Figure 8) was retrieved from GSE192742. Mouse NSCLC scRNA-seq data (related to Supplemental Figure 11) was retrieved from GSE127465.

Cell line
Human sarcoma cell line U-2 OS (ATCC, HTB-96) was purchased. U-2 OS cells were maintained in McCoy's 5a Medium (ATCC 30-2007) containing 10% FBS (ATCC, 30-2020) and 1% Anti-Anti.

Mouse
All animal work was approved by the Yale University Institutional Animal Care and Use Committee. Mice were housed at 22°C-24°C with a 12 h light/12 h dark cycle with a standard chow diet (CD, Harlan Teklad no. 2018, 18% calories from fat), a CDAA-HFD diet (Research Diets, A06071302), or a western diet (WD, Research Diets, D09100301), and water provided ad libitum. Tet3fl/fl mice were kindly provided by Anjana Rao from La Jolla Institute for Immunology. Mye-Tet3 ko mice (Lysm+/wtTet3fl/fl) were generated by crossing LysM-cre (JAX, 004781, Jackson Laboratory) and Tet3fl/fl mice. Littermate Lysmwt/wtTet3fl/fl mice were used as control (WT). For all experiments, age-matched animals were used.

Bobcat339 treatment of mice

Bobcat339 powder (Sigma-Aldrich, SML2611) was freshly dissolved in DMSO to prepare a 5 mM stock solution for in vitro studies. For tail vein injection, Bobcat339 powder was freshly dissolved in DMSO to a concentration of 50 mg/mL, sterilized by filtration through a 0.22-µm filter, and diluted with 1× PBS to a final concentration of 3 mg/kg before use. For oral gavage, Bobcat339 was prepared at a concentration of 30 mg/kg in 1× PBS with a final DMSO concentration of 20%.

Fluorescent immunohistochemistry (IHC) of tissue sections

FFPE tissue slides were deparaffinized by heating at 65°C for 60 min, followed by xylene wash 3 times 5 min each. The slides were then rehydrated in 100%, 90%, 80% and 70% ethanol for 5 min each, followed by ddH_2O wash 2 times 5 min each. For antigen retrieval, slides were heated to near boiling in sodium citrate solution (freshly prepared, pH 6.0) for 2 min, followed by incubation in a steamer (BELLA Food Steamer, Model # XJ-10102A) for 15 min. The slides were cooled at RT to below 40°C, followed by sequential wash in PBS and PBS-T (0.05% Tween 20/PBS) for 5 min each. After permeabilization in 1% Triton X-100/PBS for 15 min, slides were blocked in 5% donkey serum/PBS-T at RT for 1 h and quickly washed once with PBS-T before antibody incubation. Slides were then incubated with antibodies diluted in 2% donkey serum/PBS-T overnight at 4°C. Antibodies used are anti-CD163 (Abcam, ab156769, diluted at 1:500 for human tissue; Santa Cruz Biotechnology, sc-58965, diluted at 1:400 for mouse tissue), anti-Mac2 (Biolegend, 125401, diluted at 1:400), anti-F4/80 (Invitrogen, 17-4801-82, diluted at 1:100), anti-TET3 (GeneTex, GTX121453, diluted at 1:500 for human tissue, Millipore Sigma, ABE290, diluted at 1:500 for mouse tissue), anti-NLRP3 (Antibodies, A82453, diluted at 1:400), anti-TGFβ1 (Proteintech, 81746-2-RR, diluted at 1:200), anti-IL-1β (Proteintech, 26048-1-AP, diluted at 1:200), anti-CCL2 (R&D Systems, AF-279-NA, diluted at 15 μg/ml), anti-PD-L1 (ThermoFisher Scientific, PA5-88105, diluted at 1:600), anti-CD8α (Abcam, ab217344, diluted at 1:500), anti-GrB (ThermoFisher Scientific, MA5-11578, diluted at 1:500), and anti-Ly6G (Novus, NBP2-00441SS, diluted at 1:200). Negative controls were performed by omitting the respective primary antibodies. Of note, for CD163/PD-L1 double staining, the permeabilization step (1% Triton X-100/PBS for 15 min) was omitted. The next day, slides were washed 3 times and incubated in 0.4% Triton X-100/PBS with the secondary antibody donkey anti-Rabbit IgG Fluor 594 (Invitrogen, A-21207, diluted at 1:500), donkey anti-mouse IgG Fluor 488 (Invitrogen, A-21202, diluted at 1:500), donkey anti-Rat IgG Flour 488 (Invitrogen, A-21208, diluted at 1:500) or donkey anti-goat IgG Fluor 647 (Invitrogen, A-21447, diluted at 1:500) for 1 h at RT. The slides were covered with antifade mounting medium with DAPI (Vector laboratories, H-2000), scoped using a Keyence BZ-X700 fluorescence microscope.

Mouse tissue IHC quantification

Image analysis and fluorescent signal quantification were performed and analyzed using ImageJ. Six tissue sections per mouse were quantified, with 6-8 mice in each group. Fluorescent signal was quantified as mean fluorescence intensity (MFI) and normalized to $F4/80^+$ or $CD163^+$ macrophage area.

Mouse BMDM isolation and culture

Femur bones were collected from wild type C57BL/6J mice (000664, Jackson Laboratory). Total bone marrow cells were flushed out and collected in sterile PBS. Cell suspension was filtered through a 70 µm nylon strainer, centrifuged and resuspended in 5 mL of red blood cell lysis buffer (BioLegend, 420301) for 5 min to exclude any red blood cells. After washing away the red cell lysis buffer with PBS, the cells were centrifuged, resuspended and seeded in 6-cm dishes (RPMI-1640 with 10% FBS, 1% anti-anti, 20ng/mL M-CSF). Culture media was changed after 3 days, and the cells were cultured in fresh media with M-CSF at 20 ng/mL for an additional 4 days to induce macrophage differentiation.

Histopathology examination

Mouse liver samples were collected and fixed at 4˚C with 4% of phosphate buffered paraformaldehyde for 24 h. Fixed tissue specimens were embedded in paraffin and cut into 3-6 µM thick tissue sections for histopathological analysis. Tissue sections were stained with Hematoxylin-Eosin according to standard protocols. For Sirius Red/Fast Green staining, slides were deparaffinized by heating at 65°C for 60 min,

followed by xylene wash 3 times 5 min each. The slides were then rehydrated in 100%, 90%, 80% and 70% ethanol for 5 min each, followed by ddH2O wash 2 times 5 min each. Then they were incubated in 0.04% Fast Green (Sigma-Aldrich, F7258) in saturated picric acid for 15 min, and then incubated in 0.1% Fast Green and 0.08% Sirius Red (Sigma-Aldrich, 365546) in saturated picric acid for 30 min. After that, the slides were washed with 70%, 80%, 90% and 100% ethanol and p-xylene for 5 min each. After air dry, the slides were mounted with DPX Mounting and scoped using Keyence BZ-X700 microscope. Collagen fibers appeared red, while the non-collagen proteins were green. The NAFLD activity score (NAS) and the stage of liver fibrosis were assessed by two expert pathologists who were blinded to the treatment group.

Co-immunoprecipitation experiments

To study the interaction between TET3/STAT3 and TET3/VHL, human MDMs were seeded in 6-cm dishes at a density of 1 × 10^6 cells per dish and infected with Ad-TET3 adenovirus (Ad-h-TET3, ADV-225322, Vector Biolabs) or control Ad-GFP adenovirus (1060, Vector Biolabs) at 5 × 10^6 PFU/ml for 48 h. To test Bc effects on TET3/VHL interaction, cells were treated with Veh or Bc (50 µM) for 2 h. Cells were then washed in situ with cold PBS three times, collected by manual scraping, and pelleted by gentle centrifugation. The pellet was resuspended in cold gentle lysis buffer (GLB: 0.5% Triton X-100, 10 mM NaCl, 10 mM Tris-HCl pH 7.5, 10 mM EDTA, 1x protease inhibitor cocktail, and 1x phosphatase inhibitor cocktail) (1 ml of GLB per 2×10^6 cells) and incubated on ice for 20 min with occasional inversion. After centrifugation at 12,000 × g for 15 min at 4°C to remove insoluble materials, NaCl was added to a final concentration of 200 mM. The lysate was then transferred to a tube containing 30 µl of Anti-DYKDDDDK magnetic agarose beads (per co-IP of 2×10^6 cells) and incubated at 4°C for 4 h. The beads were washed twice with cold IP buffer, followed by three additional washes for 5 min each at 4°C. After the final wash, the residual liquid was removed, and the beads were eluted with 20 µl of 2x SDS buffer (containing 1x phosphatase inhibitor cocktail and 1x protease inhibitor cocktail) at 100°C for 5 min. The eluate was loaded (10 µl per gel well) onto a 4-15% gradient SDS gel (Bio-Rad, 456-8086). Western blot analysis was performed using anti-TET3 (Cell Signaling, 99980S, 1:1000 dilution), anti-p-STAT3 (Cell Signaling, 9145S Y705, 1:1000 dilution), or anti-VHL (Proteintech, 24756-1-AP, 1:1000 dilution) antibodies, with HRP-linked anti-rabbit IgG as the secondary antibody (Rockland, 611-1322, 1:10,000 dilution).

ChIP, hMeDIP, and MeDIP

To prepare antibodies for human MDM ChIP, 10 µL (slurry solution) ChIP-grade Protein A/G Magnetic Beads (Thermo Fisher Scientific, 26162) was washed twice in low-adhesion tubes (USA Scientific, 1415-2500) with 1 mL binding buffer (0.2% Tween-20 in PBS), followed by incubation on a rotator with 2.5 µg anti-TET3 (Active Motif, 61395), 2.5 µg anti-STAT3 (Proteintech, 10253-2-AP), 2.5 µg anti-H3K4me3 (Cell Signaling Technology, 9751S), or 2.5 µg normal rabbit IgG (Cell Signaling Technology, 2729S) in 200 µL binding buffer at 4°C overnight. Antibody-bound beads were washed twice with 1 mL binding buffer and once with dilution buffer (0.01% SDS, 1.1% Triton X-100, 1.2 mM EDTA, 16.7 mM Tris-HCl at PH 8.0, 167 mM NaCl), resuspended in 50 µL dilution buffer and kept on ice until use.

Human MDMs were plated in 6-cm culture dishes at 1x10^6 cells/dish and infected with Ad-GFP or Ad-TET3 at 5 × 10^6 PFU/ml for 16 h. Cells were cross-linked in culture media at RT for 10 min in 1% paraformaldehyde (32% stock, Electron Microscopy Science, 15714), followed by addition of glycine buffer (150 mM final concentration) to quench cross-linking for 5 min. Cross-linked cells were washed 3 times with cold PBS and harvested in 1 mL cold cell lysis buffer (50 mM Tris-HCl at pH 8.0, 140 mM NaCl, 1 mM EDTA, 10% glycerol, 0.5% NP-40, 0.25% Triton X-100) by scraping, and the cell suspensions were transferred to new low-adhesion tubes followed by incubation at 4°C for 15 min to lyse the plasma membrane. Nuclei were pelleted by centrifugation at 2,000 g for 10 min at 4°C and resuspended in 150 µL cold nuclear lysis buffer (10 mM Tris-HCl at PH 8.0, 0.5 mM EGTA, 1 mM EDTA, 0.2% SDS), followed by rotation at 4°C for 20 min. Chromatin was sheared to produce 200–700 bp chromatin fragments using a sonifier (Branson 150), with a setting of 5 pulses of 10 sec each at 30% amplitude followed by a 60 sec rest period on ice between each pulse. Samples were centrifuged at 16,000 g for 10 min at 4°C to remove insoluble materials and the resulting supernatant was subjected to a 4-fold dilution. Diluted chromatin solutions (5% of total) were saved as input samples and kept at

-80 ℃ until use. To perform ChIP, 250 µL diluted chromatin (~5x10^5 cells per ChIP) was added to each tube containing antibody-bound beads and incubation on a rotator was carried out overnight at 4°C. Beads were washed 8 times with 1 mL cold wash buffer (100 mM Tris-HCl at pH 8.0, 500 mM NaCl, 1 % deoxycholic acid, 1% NP-40) by rotating at 4°C for 5 min and standing on the magnetic column for 1 min each. After the last washing with wash buffer, beads were washed once with cold TE buffer (50 mM Tris-HCl at pH 8.0, 10 mM EDTA) by rotating at 4°C for 5 min. Beads were eluted with 85 µL elution buffer (50 mM Tris-HCl at pH 8.0, 10 mM EDTA, 1% SDS) by agitation in a thermomixer (Thermo Scientific) at 65°C, 1500 rpm for 15 min. Elution was repeated once, and the 2 eluants were combined. RNase (10 µg) was added to each sample (including the input samples), followed by incubation at 37℃ for 1 h. Proteinase K (200 µg) dissolved in 120 µL TE buffer was then added to each sample, and the tubes were incubated at 65℃ overnight. Samples were purified using the QIAquick PCR Purification Kit (QIAGEN, 28104) and eluted in a volume of 50 µL. ChIP-purified DNA levels were determined by qPCR. Primers used are listed in the Supplementary Table 1. The relative enrichments of the DNA regions were calculated using the percentage input method as previously described (*1*). For Sttatic inhibition ChIP, human MDMs were plated in 6-cm culture dishes at 2x10^6 cells/dish and infected with Ad-TET3 at 5 × 10^6 PFU/ml for 20 h. Sttatic (HY-13818, MedChemExpress) or Veh (DMSO) was added to a final concentration of 5 µM and incubation was carried out for 45 min, followed by ChIP using anti-TET3 (Active Motif, 61395).

To perform NLRP3 ChIP in human U-2 OS sarcoma cell line (HTB-96, ATCC), cells were seeded in 10-cm dishes at a density of 1 × 10^7 cells per dish, and infected with Ad-GFP or Ad-TET3 at 5 × 10^5 PFU/ml for 16 h. To prepare antibodies for Flag-TET3, STAT3 and H3K4me3 ChIP, 50 µl Anti-DYKDDDDK magnetic agarose beads, 5 µg anti-STAT3, 5 µg anti-H3K4me3 (Cell Signaling Technology, 9751S), or 5 µg normal rabbit IgG per ChIP of 1 × 10^7 cells were used, respectively.

To perform hMeDIP or MeDIP, MDMs were infected with Ad-GFP or Ad-TET3 for 16 h, and the DNA was collected using Zymo Quick-DNA Microprep kit (Zymo D3021). DNA was sheared to produce 200-700 bp DNA fragments using a sonifier (Branson 150), with a setting of 4 pulses of 12 sec each at 25% amplitude followed by a 60-sec rest period on ice between each pulse. The sonicated DNA was subjected to hMeDIP using the EpiQuick Hydroxymethylated DNA Immunoprecipitation Kit (EpigenTek, P1038-24) or EpiQuick MeDIP Ultra Kit (EpigenTek, P1052-24) according to manufacturers' protocols. Immunoprecipitated DNA levels were determined by qPCR.

Western blot analysis
To extract proteins from cultured macrophages, cells were homogenized in situ using a pipette tip in 2x SDS-sample buffer with 10% β-mercaptoethanol at RT followed by heating at 100 °C for 5 min with occasional vortexing. To extract proteins from mice liver tissues, frozen tissue samples (~50 mg) were homogenized in 500 ml of tissue lysis buffer (15% SDS, 75 mM Tris-HCl, pH 7.4, 1x Protease inhibitor cocktail [Thermo, 78438], 1x Phosphatase inhibitor cocktail [Thermo, 78427], 5% b-mercaptoethanol) using a BeadBug6 Microtube homogenizer (Benchmark) set at speed 3600, 20s on and 20s off for 6 cycles. The lysate was cooled down on ice for 10 min, followed by centrifugation at 12,000 x g at 4 ℃ for 7 min to remove insoluble materials. Supernatant was transferred to a new tube and glycerol (final concentration 20%) and Bromophenol (for tracking purpose during gel running) were added. Samples were then heated at 100°C for 5 min with occasional vortexing, aliquoted, and stored at -80 °C until use. Cell and tissue samples were loaded at 5-12 µL/well onto a 4-15% gradient SDS gels (Bio-rad, 456-8086), transferred to nitrocellulose membranes, followed by Western blot analysis. The antibodies used were anti-TET3 (Cell Signaling, 99980S, diluted at 1:1000), anti-TET2 (Proteintech, 21207-1- AP, diluted at 1:2000), anti-TET1 (Abcam, ab272900, diluted at 1:500), anti-NLRP3 (Proteintech, 68102-1-Ig, diluted at 1:1000), anti-TGFβ1 (Abcam, ab215715, diluted at 1:1000), anti-a-SMA (Invitrogen, 14-9760-82, diluted at 1:1000), anti-COL1A (Santa Cruz Biotechnology, sc-59772, diluted at 1:1000), anti-VHL (Proteintech, 24756-1-AP, diluted at 1:1000 for human; Invitrogen, PA5-27322, diluted at 1:1000 for mouse), and HRP-conjugated anti-GAPDH (Proteintech, HRP-60004, dilution 1:10000). The

secondary antibody was HRP-linked Anti-rabbit IgG (Rockland, 611-1322, dilution 1:10,000) or HRP-linked Anti-mouse IgG (Proteintech, SA00001-1, dilution 1:10,000)

qRT-PCR
Total RNAs were extracted from cells using PureLink RNA Mini Kit. 0.5-1 μg total RNA was reverse transcribed to cDNA in a reaction volume of 10-20 μL using PrimeScript RT Reagent Kit. Real-time quantitative PCR was performed in a 15 μL reaction volume containing 0.5–1 μL of cDNA using SsoAdvanced Universal SYBR Green Supermix in a Bio-Rad iCycler. Gene expression levels were normalized against RPLP0. The specific PCR primers were summarized in Supplementary table 2.

Inflammasome activity assays
Human MDMs or murine BMDMs were infected with Ad-TET3 or Ad-GFP at 5×10^7 PFU/mL for 24 h. The next day, the medium was changed to wash away the adenoviruses. Cells were then primed with LPS at 250 ng/mL for 4 h, followed by addition of nigericin at 20 μM for 1 h (for Caspase1 assay) or 1.5 h (for IL-1β assay). Caspase 1 activity and IL-1β concentration were measured using CaspaseGlo 1 inflammasome assay kit (Promega, G9951), Lumit™ Human IL-1β Immunoassay kit (Promega, W6010, for human), or LumitTM Mouse IL-1β Immunoassay kit (Promega, W7010, for mouse) according to manufacturer's protocols.

Measurements of CCL2 and IL-1β protein level in primary macrophages
The ELISA kit for CCL2 (R&D Systems, DCP00) and IL-1β (Elabscience, E-EL-H0149c) were used according to the manufacturers' protocols. For CCL2 measurements, primary macrophages were seeded in 24-well plates at a density of 90% with 300 μl of culture medium per well. Cell culture supernatants were collected and centrifuted to remove debris before ELISA analysis. For IL-1β measurements, cells at 1×10^6 were detached using Trypsin-EDTA (0.25%) followed by centrifugation at 1000 x g for 5 min. The supernatants were discarded and cell pellets were resuspended in 150 μL PBS containing protease inhibitors. The resulting cell suspension was incubated in liquid nitrogen for 30 min followed by rapid thawing in a 37°C water bath. Repeat the above steps three times. The cell lysate was cleared by centrifugation at 1500 x g for 10 min at 4°C to remove insoluble materials. CCL2 and IL-1β protein concentrations were presented after normalization against numbers of viable cells.

AlphaScreen assay
The assay was conducted in a total reaction volume of 50 μL using HBS-P buffer (Cytiva, BR100368) with 0.5% BSA as the base. Initially, 9 μL of HBS-P buffer was added to each well, followed by sequential addition of 10 μL of α-ketoglutarate (α-KG) solution (final concentration: 125 μM), 10 μL of ammonium ferrous sulfate solution (final concentration: 125 μM), 10 μL of TET protein (final concentration: 1 nM), and 10 μL of VHL protein (final concentration: 1 nM). Next, 1 μL of test compounds (Bobcat 339 in DMSO, dissolved in the specified formula) was added. The reaction mixture was gently mixed using a pipette and incubated at room temperature for 1 h. Subsequently, 5 μL of acceptor beads (10 μg/mL, diluted in HBS-P buffer) was added to each well. The mixture was gently pipetted to ensure uniformity and incubated for an additional 1 h at RT. Then, 5 μL of donor beads (10 μg/mL, diluted in HBS-P buffer) was added to each well, followed by gentle pipetting to mix thoroughly. The plate was incubated at room temperature for 30 min, and the signal was measured using a PHERAstar plate reader according to the manufacturer's instructions. The following reagents were used: TET1 (Abcam, ab271753), TET2 (Abcam, ab271754), TET3 (Bioscience, 50163), VHL (Creative BioMart, VHL-29832TH), and the AlphaScreen kit (Revvity, 6760613C).

TUNEL Assays
TUNEL staining of mouse liver FFPE slides was performed using One-step TUNEL Assay Kit (E-CK-A320 Elabscience) to detect apoptotic cells according to the manufacturer's instructions. Images were captured using a Keyence BZ-X700 fluorescence microscope.

Blood and liver chemistry

Blood samples were collected in EDTA tubes (Microtainer with K2EDTA, BD, 365974) by cardiac puncture of terminally anesthetized animals. The tubes were centrifuged at 2,000 x g at 4 ^{0}C for 20 min, and plasma was collected and stored at -80 ^{0}C until use. Blood chemistry was measured according to the manufacturer's instructions. Kit used to measure alanine transaminase (EALT-100) and Aspartate Transaminase (EASTR-100) were purchased from Bioassay Systems. Measurements of liver tissue triglycerides and hydroxyproline contents were performed using Triglyceride Assay Kit (Abcam, Ab65336) and Hydroxyproline Assay Kit (Sigma-Aldrich, MAK008-1KT), respectively.

Glucose tolerance test
GTT were performed following 16-h overnight fasting. Each animal received an i.p. injection of 2 g/kg of glucose (Sigma-Aldrich, G5767) in sterile saline. Blood glucose concentrations were measured using Contour next blood glucose meter (Ascensia Diabetes Care) via tail vein bleeding at the indicated time points after injection.

Flow cytometry
Total bone marrow cells were harvested from femur bones of mice into the PBS. Cell suspension was filtered through a 70 μm nylon strainer, centrifuged and resuspended in 5 mL of red blood cell lysis buffer for 5 min (BioLegend, 420301) to exclude any red blood cells. The cells were resuspended in PBS and stained by antibodies for 15 min in the dark at RT. The antibodies used were CD11b-PE (diluted at 1:100, BioLegend, 101208), NK1.1-APC (diluted at 1:50, BioLegend, 108710), Ly6C-PerCP (diluted at 1:100, BioLegend, 128028) and Ly6G-FITC (diluted at 1:200, BioLegend, 127606). Data were acquired using BD FACSCalibur flow cytometry system and analysis was performed using Flowjo V10.

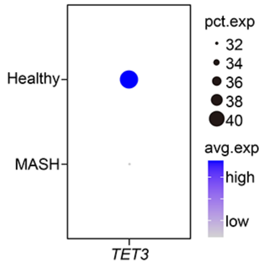

Supplemental Figure 1
Dot plots showing TET3 expression levels in non-KCs from Healthy (n=8) and MASH (n=7 patients) liver samples. Refer to Supplemental Table 1 for detailed patient sample information.

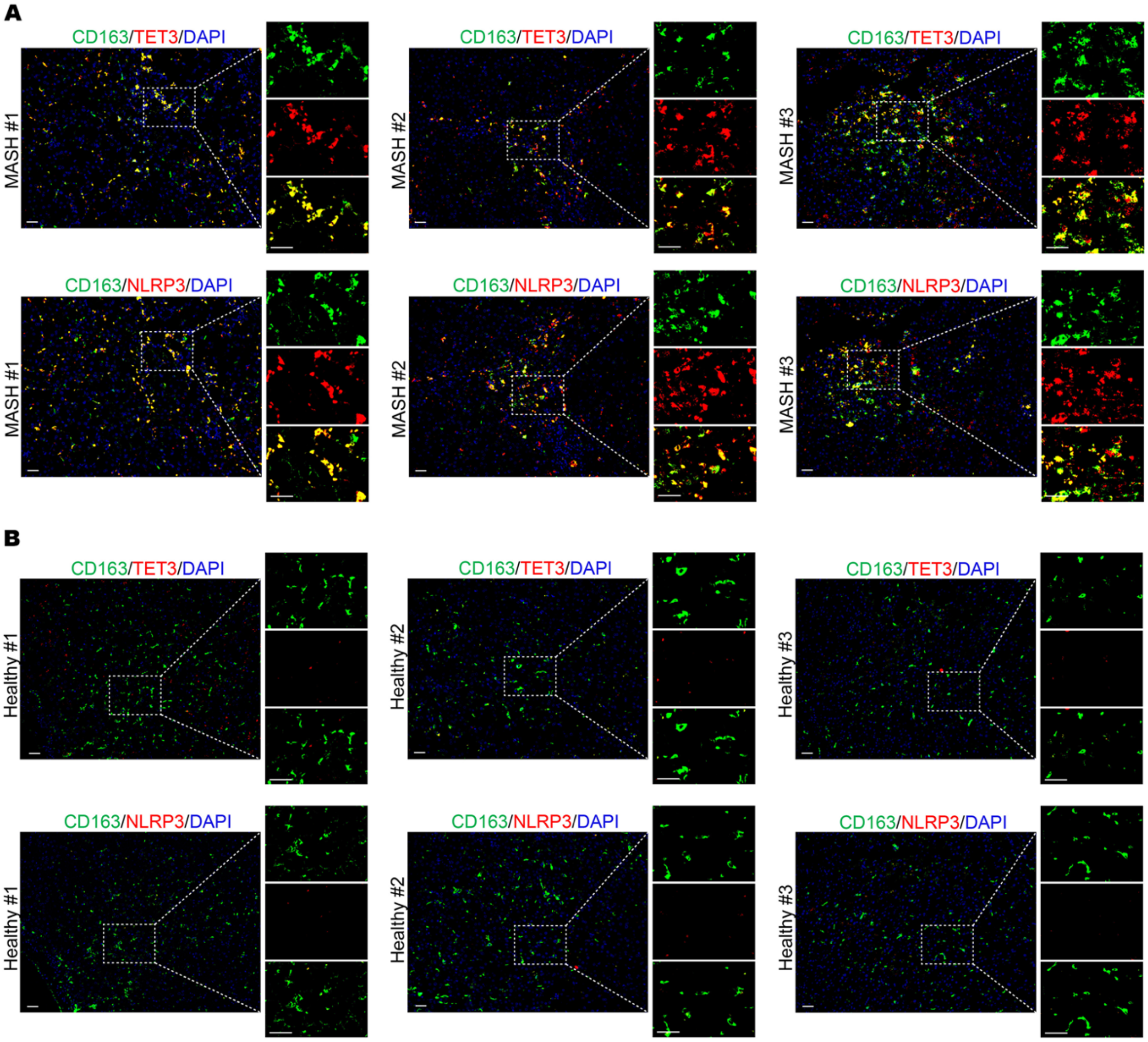

Supplemental Figure 2
(**A**) IHC images of TET3 (red, top panels) and NLRP3 (red, bottom panels) co-stained with CD163 (green) and nuclei (blue) of liver tissue sections from 3 different MASH patients. The top panels are serial sections of the bottom panels. (**B**) IHC images of TET3 and NLRP3 co-stained with CD163 and nuclei of liver tissue sections from 3 healthy controls. The top panels are serial sections of the bottom panels. Scale bar: 10 μm. Refer to Supplemental Table 1 for detailed patient sample information.

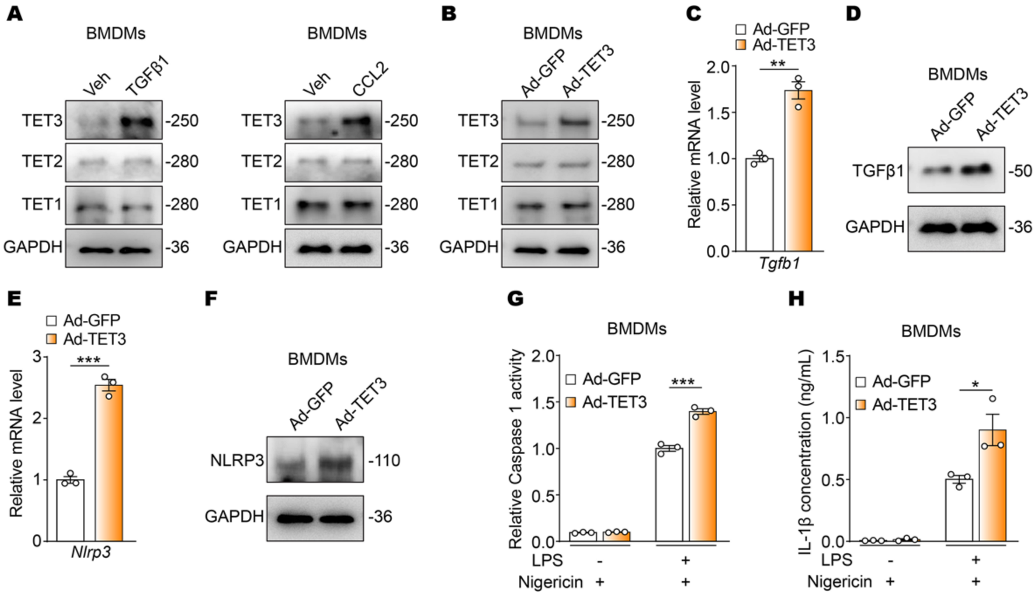

Supplemental Figure 3

(**A**) Mouse BMDMs were treated with vehicle (Veh), TGFβ1 (30 ng/ml), or CCL2 (100 ng/ml). Proteins were isolated at 24, followed by western blot analysis for TET3, TET2 and TET1. GAPDH was used as a loading control. (**B**) Western blots of TET3, TET2 and TET1 in BMDMs infected with Ad-GFP or Ad-TET3 for 24 h. (**C** and **D**) TGFβ1 expression in BMDMs infected with Ad-GFP or Ad-TET3, assessed by qRT-PCR (**C**, RNA harvested at 24 h) and western blot (**D**, protein harvested at 24 h). (**E** and **F**) NLRP3 expression in BMDMs infected with Ad-GFP or Ad-TET3, assessed by qRT-PCR (**E**, RNA harvested at 12 h) and western blot (**F**, protein harvested at 24 h). (**G** and **H**) BMDMs seeded in 96 well plates were infected with Ad-GFP or Ad-TET3 for 24 h, followed by priming with or without LPS (250 ng/ml) for 4 h. Cells were then treated with Nigericin (20 μM) for 1 h, followed by Caspase 1 activity (**G**) and IL-1β detection (**H**). All data represent the mean ± SEM, *P < 0.05, **P < 0.01 and ***P < 0.001, by 2-tailed Student's t-test. Western blot data are representative of 2 biological repeats.

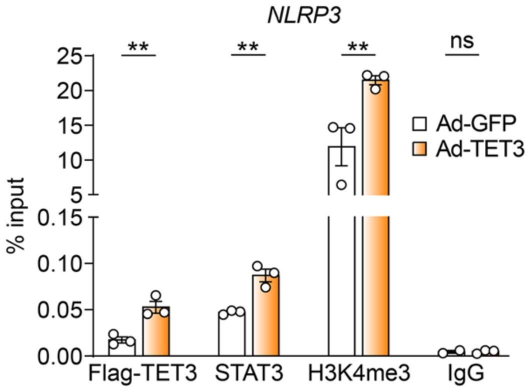

Supplemental Figure 4

Human sarcoma U-2 OS cells were infected with Ad-GFP or Ad-TET3 for 16 h, followed by ChIP-qPCR analysis using $1x10^7$ cells per ChIP. Anti-Flag antibody was used to ChIP Flag-TET3 expressed from Ad-TET3 with its associated *NLRP3* promoter region. Data represent the mean ± SEM, **P < 0.01 by 2-tailed Student's t-test. ns, not statistically significant.

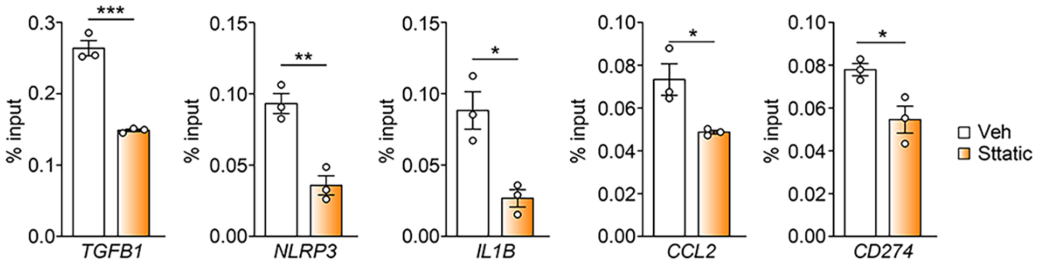

Supplemental Figure 5
Human MDMs were plated in 6-cm culture dishes at 2x10^6 cells/dish and infected with Ad-TET3 at 5 × 10^6 PFU/ml for 20 h. Sttatic or Veh (DMSO) was added to a final concentration of 5 μM and incubation was carried out for 45 min, followed by ChIP using TET3 antibody. Data represent the mean ± SEM, *P < 0.05, **P < 0.01, and ***P < 0.001 by 2-tailed Student's t-test.

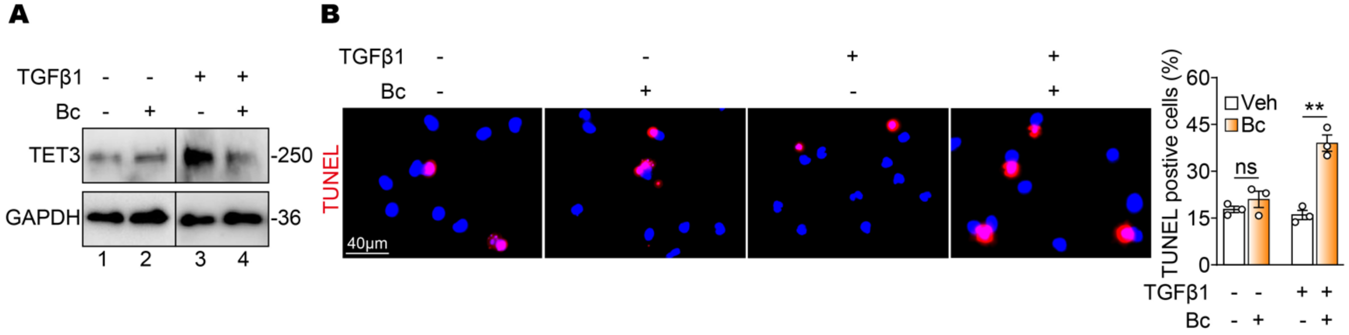

Supplemental Figure 6
Disease-induced Toe-Macs are vulnerable to TET3 reduction. Peritoneal macrophages were isolated from WT mice, followed by exposure to Veh (-) or 30 ng/ml of TGFβ1 (+). Forty-eight hours later, media were replaced with fresh media without (-) or with (+) Bc at a final concentration of 10 μM. Incubation was carried out for 24 h before protein and TUNEL analyses. Representative western blots (**A**) and photomicrographs and corresponding statistical analysis (**B**) are shown. Data represent the mean ± SEM. n = 3 randomly selected areas per group (**B**). ***P* < 0.01, by 2-tailed Student's *t* test. ns, not statistically significant.

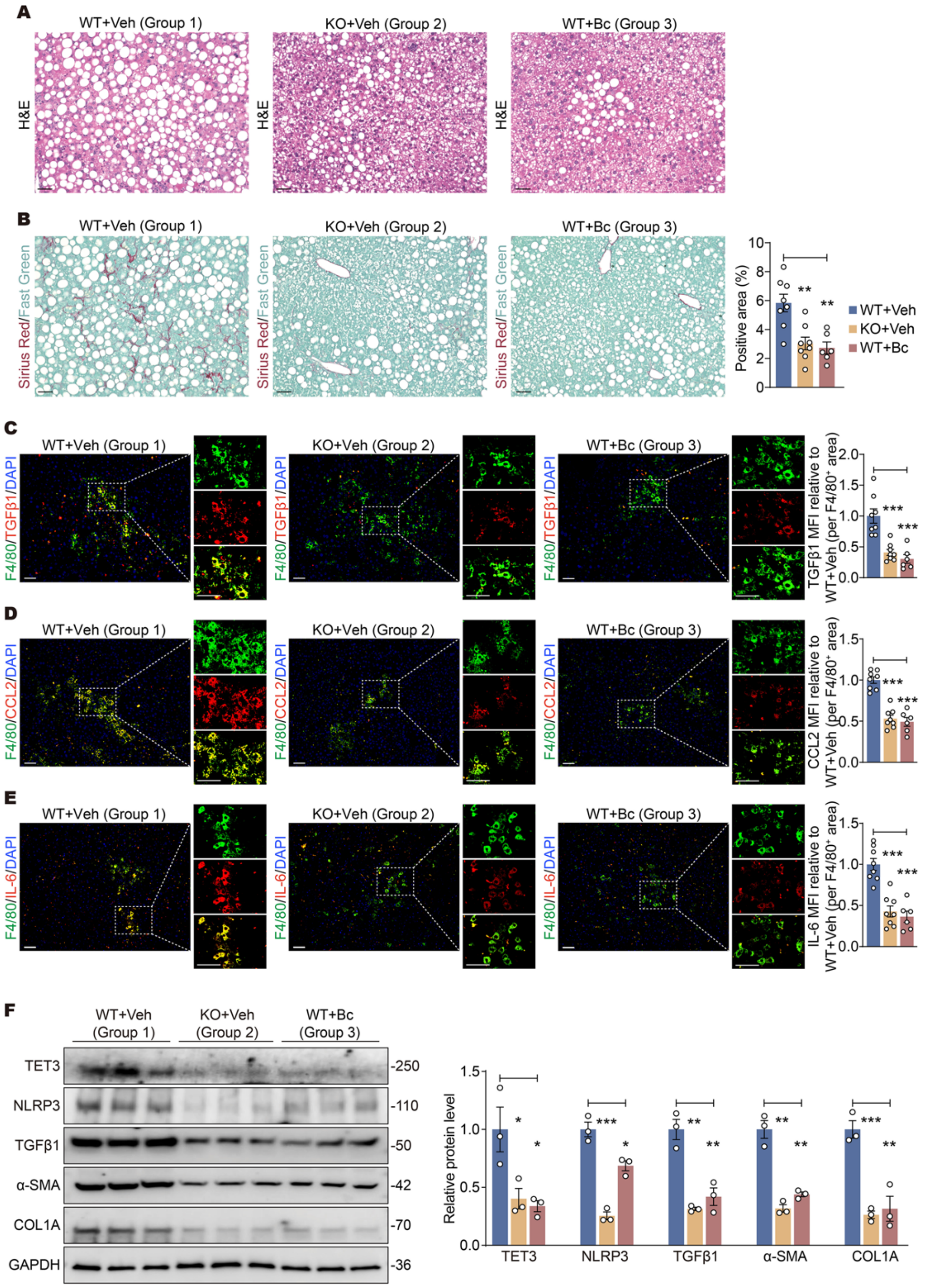

Supplemental Figure 7

Mice were treated as in Figure 5**A**.

(**A**) Representative H&E-stained liver images from mice (n = 6-8 mice per group) treated as indicated. (**B**) Representative Sirius Red/Fast Green-stained liver images and fibrosis quantification (%) from mice (n = 6-8 mice per group) treated as indicated. (**C**) Representative immunostaining of TGFβ1 (red) and F4/80$^+$ macrophages (green) and quantification of macrophage TGFβ1 MFI in liver tissue sections. (**D**) Representative immunostaining of CCL2 (red) and F4/80$^+$ macrophages (green) and quantification of macrophage CCL2 MFI in liver tissue sections. (**E**) Representative immunostaining of IL-6 (red) and F4/80$^+$ macrophages (green) and quantification of macrophage IL-6 MFI in liver tissue sections. (**F**) Representative immunoblots for TET3, NLRP3, TGFβ1, α-SMA and COL1A of liver tissues and quantification from mice (n = 3 mice per group) treated as indicated. All data represent the mean ± SEM. *P < 0.05, **P < 0.01 and ***P < 0.001 indicated the statistic differences from WT+Veh (Group 1), by 1-way ANOVA with Tukey's post-test. Scale bar: 25 μm.

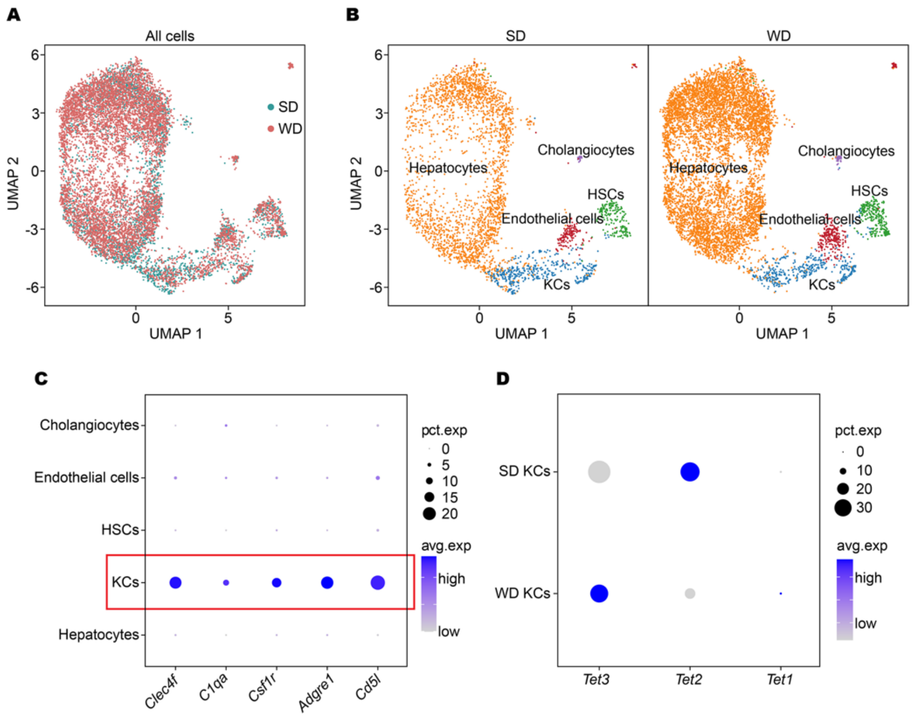

Supplemental Figure 8
(**A** and **B**) Dimension reduction plots showing all liver cells from mice fed on standard diet (SD) or western diet (WD) for 24 weeks. (**C**) Dot plot showing annotated KCs expressing typical KC markers. (**D**) Dot plot showing expression levels of *Tet3*, *Tet2* and *Tet1* in KCs from SD and WD mice.

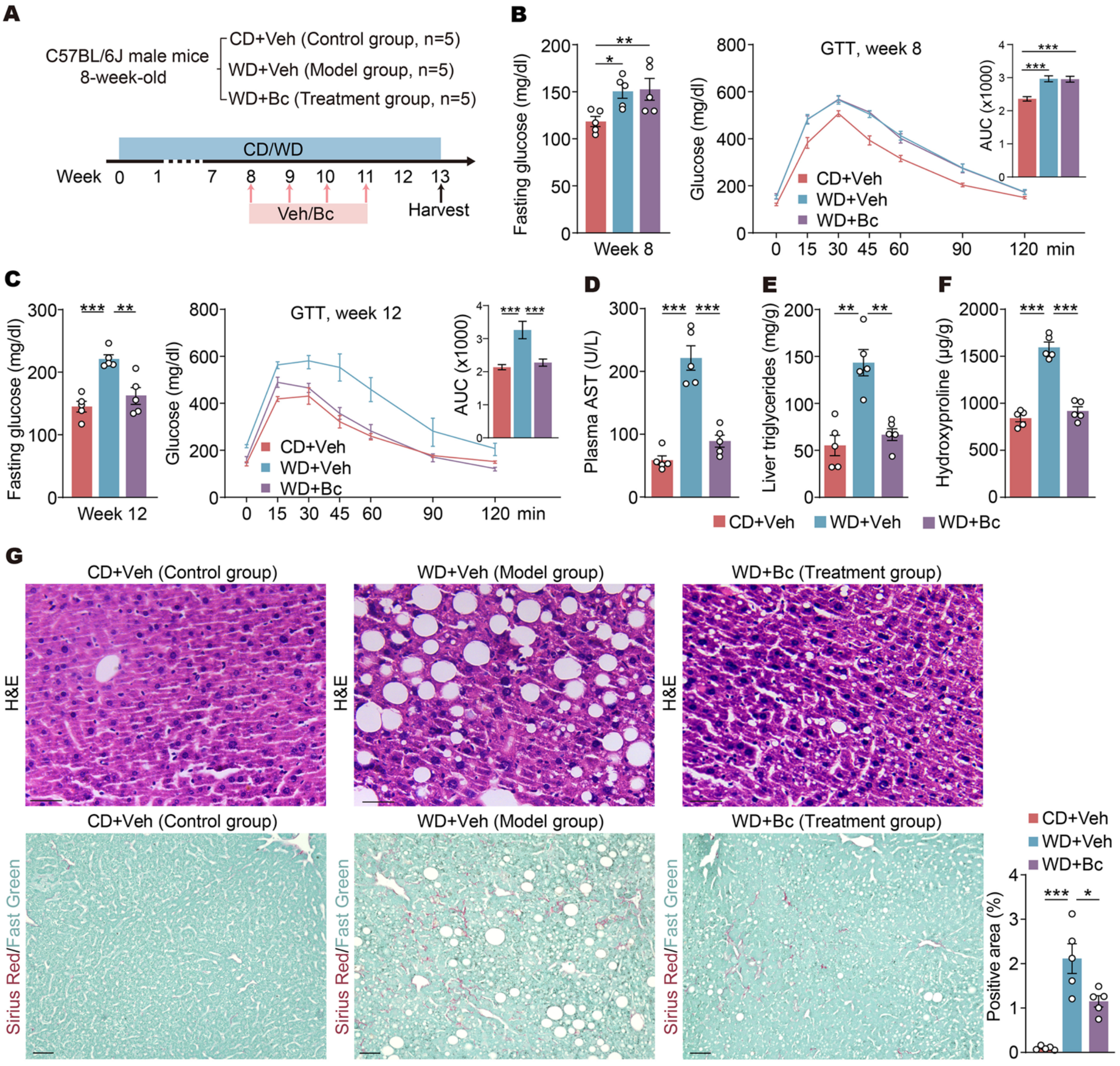

Supplemental Figure 9

(**A**) Schematic of experimental design. (**B** and **C**) Results of fasting glucose and glucose tolerance test (GTT) performed at week 8 (**B**) and 12 (**C**), respectively. (**D**) Plasm aspartate aminotransferase (AST), (**E**) Liver tissue triglycerides, and (**F**) Hepatic hydroxyproline content measured at week 13. (**G**) Representative Sirius Red/Fast Green- and H&E-stained liver images from mice treated as indicated. n = 5 mice per group. Each dot represents an animal. All data represent the mean ± SEM. *P < 0.05, **P < 0.01 and ***P < 0.001 indicated the statistic differences from Model group, by 1-way ANOVA with Tukey's post-test. AUC, area under the curve. Scale bar: 25 μm.

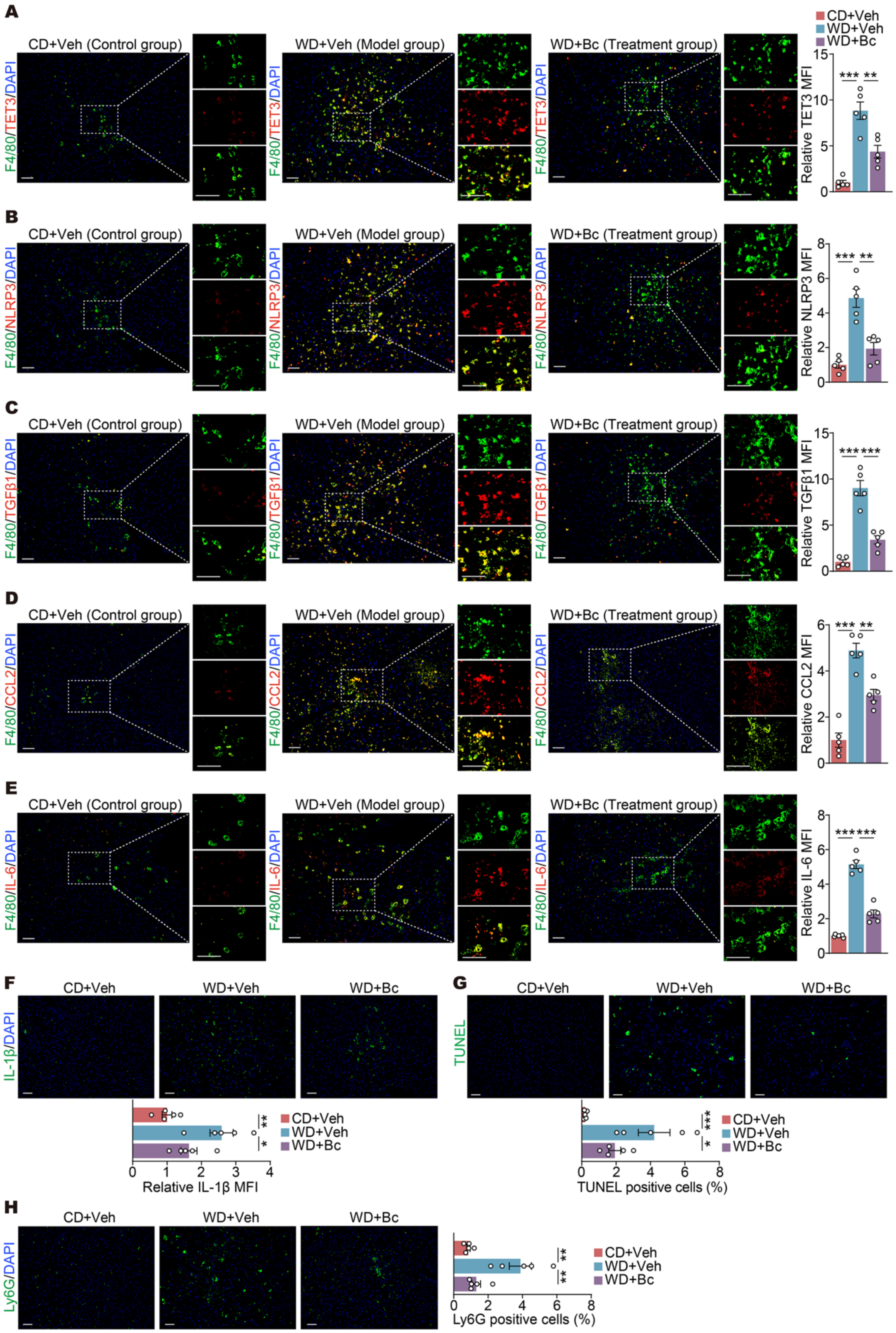
A
CD+Veh (Control group)
WD+Veh (Model group)
WD+Bc (Treatment group)
F4/80/TET3/DAPI
CD+Veh
WD+Veh
WD+Bc
Relative TET3 MFI
B
F4/80/NLRP3/DAPI
Relative NLRP3 MFI
C
F4/80/TGFβ1/DAPI
Relative TGFβ1 MFI
D
F4/80/CCL2/DAPI
Relative CCL2 MFI
E
F4/80/IL-6/DAPI
Relative IL-6 MFI
F
IL-1β/DAPI
Relative IL-1β MFI
G
TUNEL
TUNEL positive cells (%)
H
Ly6G/DAPI
Ly6G positive cells (%)

Supplemental Figure 10
(**A**) Representative immunostaining of TET3 (red) in F4/80+ macrophages (green) and quantification of macrophage TET3 MFI in liver tissue sections. (**B**) Representative immunostaining of NLRP3 (red) in F4/80+ macrophages (green) and quantification of macrophage NLRP3 MFI in liver tissue sections. (**C**) Representative immunostaining of TGFβ1 (red) in F4/80+ macrophages (green) and quantification of macrophage TGFβ1 MFI in liver tissue sections. (**D**) Representative immunostaining of CCL2 (red) in F4/80+ macrophages (green) and quantification of macrophage CCL2 MFI in liver tissue sections. (**E**) Representative immunostaining of IL-6 (red) in F4/80+ macrophages (green) and quantification of macrophage IL-6 MFI in liver tissue sections. (**F**) Representative immunostaining of IL-1β (green) and quantification of IL-1β MFI in liver tissue sections. (**G**) Representative TUNEL staining and quantification of TUNEL+ cells in liver tissue sections. (**H**) Representative immunostaining of Ly6G (green) and quantification of Ly6G+ cells in liver tissue sections. n = 5 mice per group. All data represent the mean ± SEM. *P < 0.05, **P < 0.01 and ***P < 0.001 indicated the statistic differences from WD+Veh group, by 1-way ANOVA with Tukey's post-test. Scale bar:25 μm.

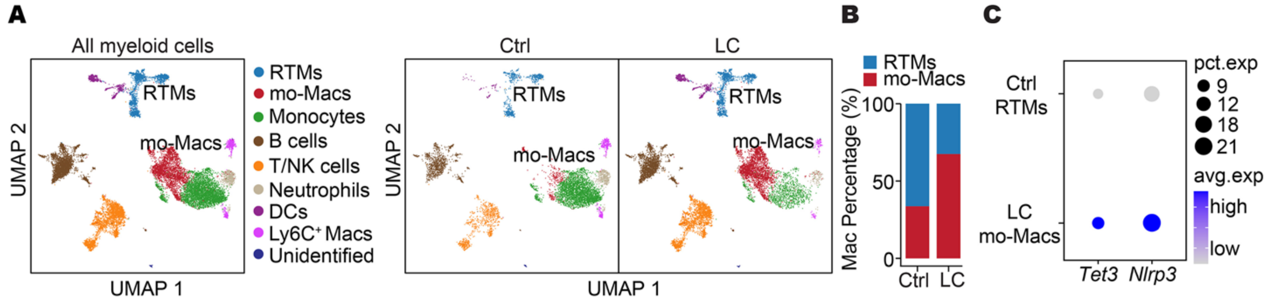

Supplemental Figure 11
(**A**) Dimension reduction plots showing immune cells from healthy control mice (Ctrl) and lung cancer bearing mice (LC). (**B**) Bar plot showing percentages of RTMs and mo-Macs in Ctrl and LC groups. (**C**) Dot plot showing expression levels of *Tet3* and *Nlrp3* in Ctrl RTMs and LC mo-Macs.

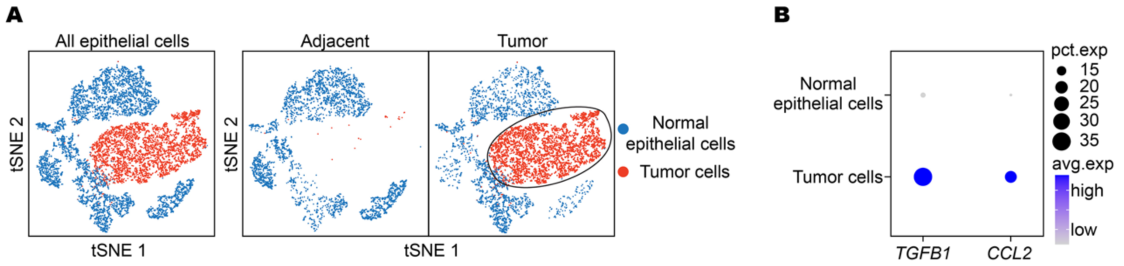

Supplemental Figure 12
(**A**) Dimension reduction plots showing all epithelial cells from Adjacent and Tumor tissues. (**B**) Dot plot showing expression levels of *TGFB1* and *CCL2* in normal epithelial cells and tumor cells. Refer to Supplemental Table 1 for detailed patient sample information (GSE131907).

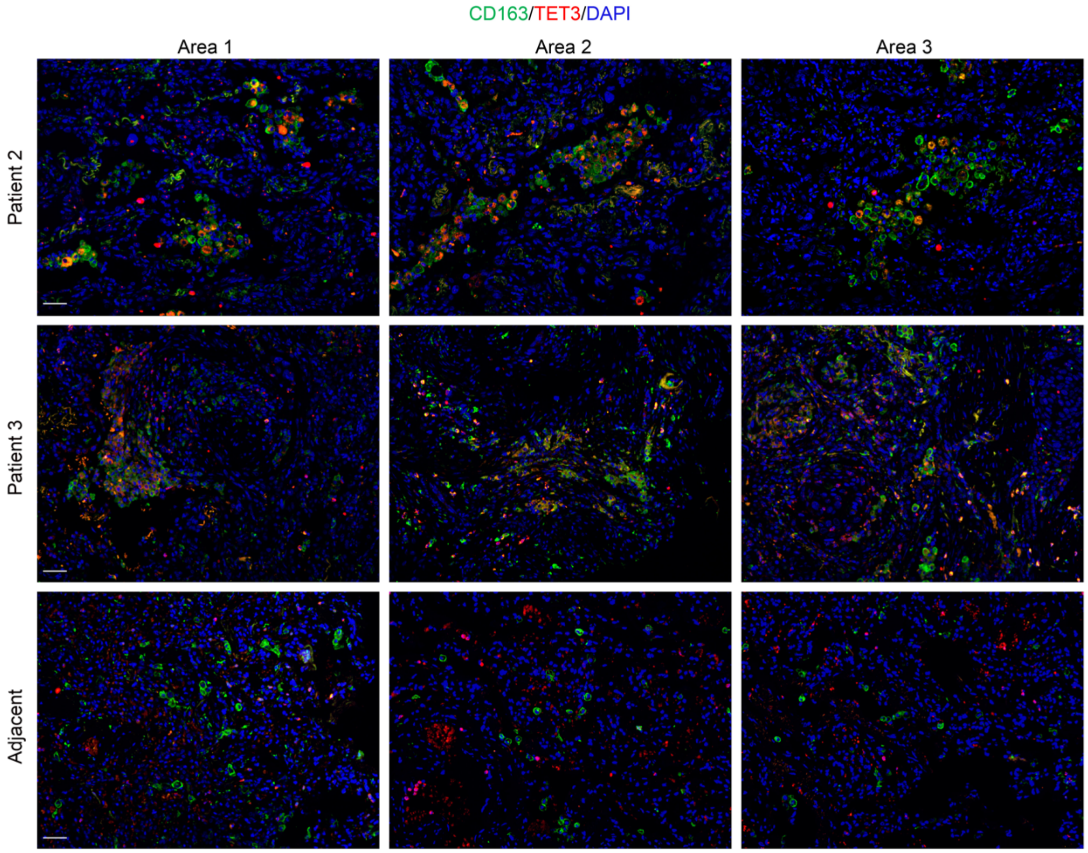

Supplemental Figure 13
Representative immunofluorescence staining of CD163 (green) and TET3 (red) in human NSCLC tumor tissues and adjacent normal tissues. Scale bar: 50 µm. Refer to Supplemental Table 1 for detailed patient sample information. Note, the “Adjacent” image on the left is also shown in Figure 6D.

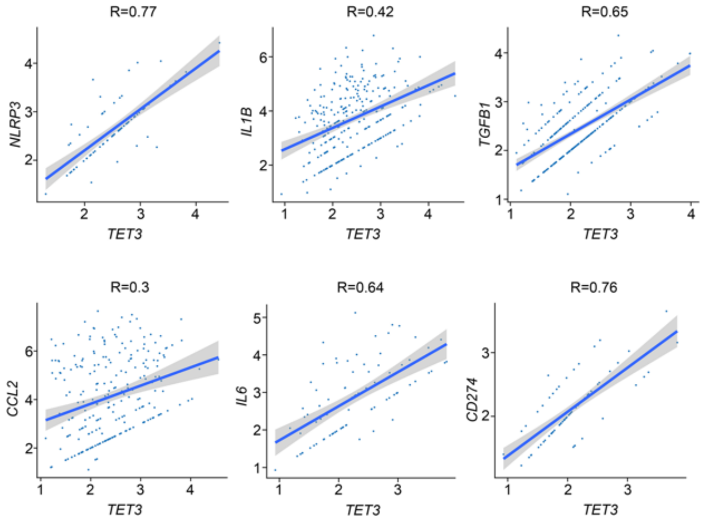

Supplemental Figure 14
Scatter plots showing positive correlations between *TET3* expression and those of *NLRP3, IL1B, TGFB1, CCL2, IL6* and *CD274* in macrophages from human lung tissue. Refer to Supplemental Table 1 for detailed patient sample information (GSE131907).

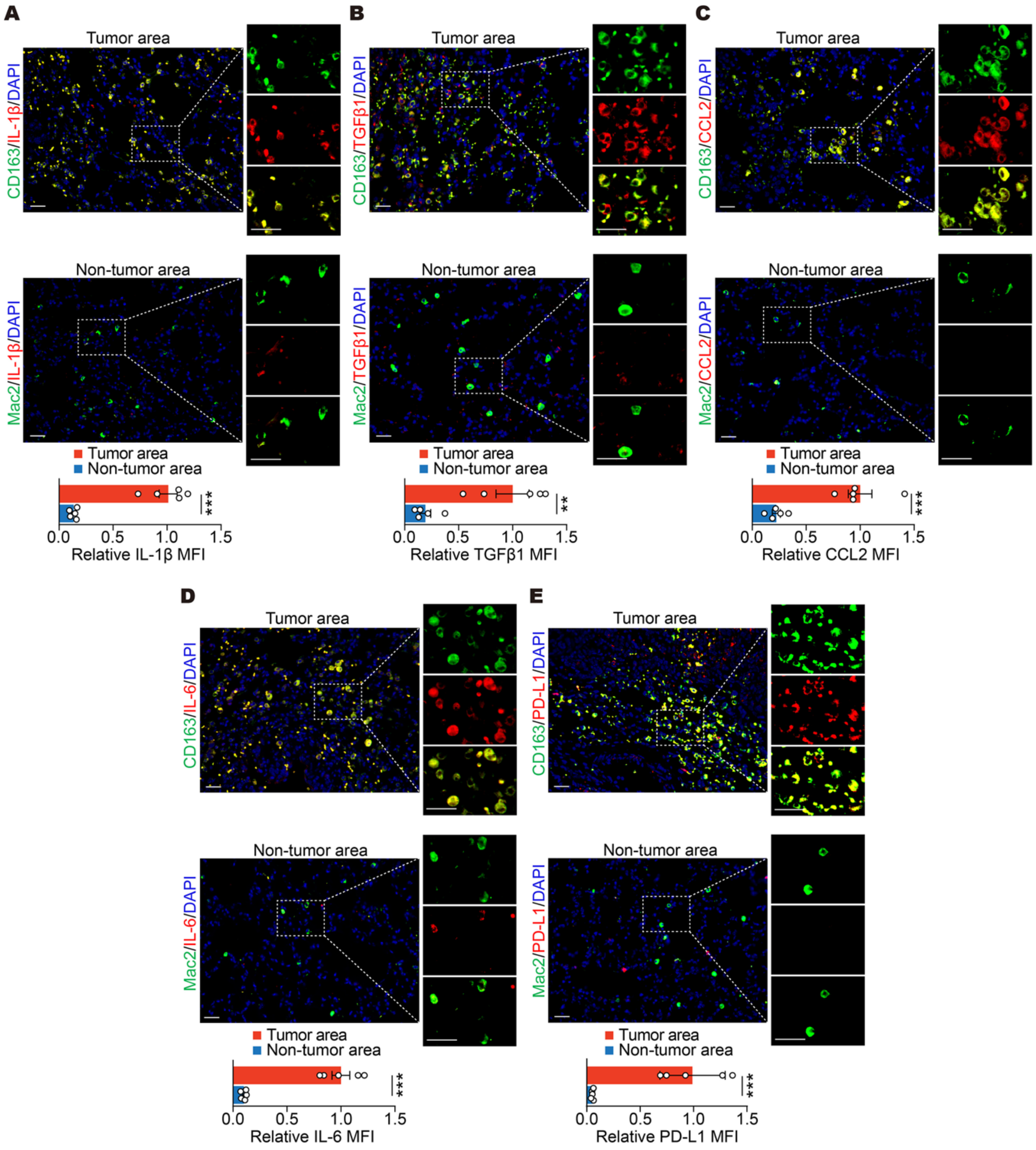

Supplemental Figure 15

(**A**) Representative immunostaining of IL-1β (red) in CD163^{+} macrophages (green) in tumor areas (up), IL-1β (red) in Mac2^{+} macrophages (green) in non-tumor areas (middle), and quantification of macrophage IL-1β MFI in tumor and non-tumor areas (low).
(**B**) Representative immunostaining of TGFβ1 (red) in CD163^{+} macrophages (green) in tumor areas (up), TGFβ1 (red) in Mac2^{+} macrophages (green) in non-tumor areas (middle), and quantification of macrophage TGFβ1 MFI in tumor and non-tumor areas (low).
(**C**) Representative immunostaining of CCL2 (red) in CD163^{+} macrophages (green) in tumor areas (up), CCL2 (red) in Mac2^{+} macrophages (green) in non-tumor areas (middle), and quantification of macrophage CCL2 MFI in tumor and non-tumor areas (low).
(**D**) Representative immunostaining of IL-6 (red) in CD163^{+} macrophages (green) in tumor areas (up), IL-6 (red) in Mac2+ macrophages (green) in non-tumor areas (middle), and quantification of macrophage IL-6 MFI in tumor and non-tumor areas (low).
(**E**) Representative immunostaining of PD-L1 (red) in CD163^{+} macrophages (green) in tumor areas (up), PD-L1 (red) in Mac2^{+} macrophages (green) in non-tumor areas (middle), and quantification of macrophage PD-L1 MFI in tumor and non-tumor areas (low).
n = 5 mice per group. All data represent the mean ± SEM. **P < 0.01 and ***P < 0.001 indicated the statistic differences from WT+Veh group, by 2-tailed Student's t-test. Scale bar: 50 μm.

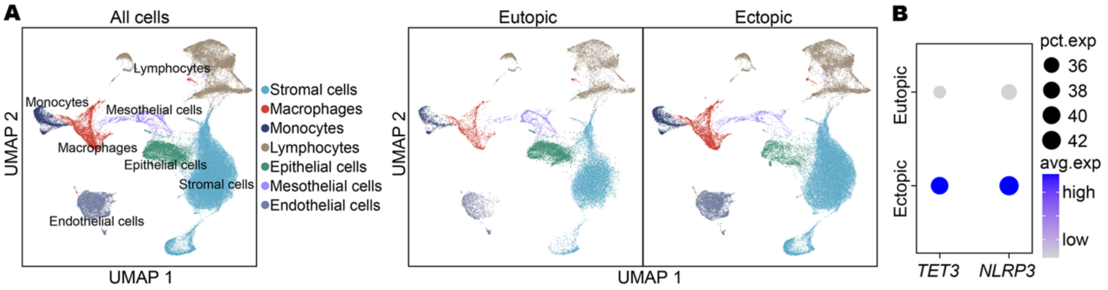

Supplemental Figure 16
(**A**) Dimension reduction plots showing all cells from ectopic and matched eutopic samples from endometriosis patients. (**B**) Dot plot showing expression *TET3* and *NLRP3* in macrophages from ectopic and matched eutopic samples. Refer to Supplemental Table 1 for detailed patient sample information (GSE17964).

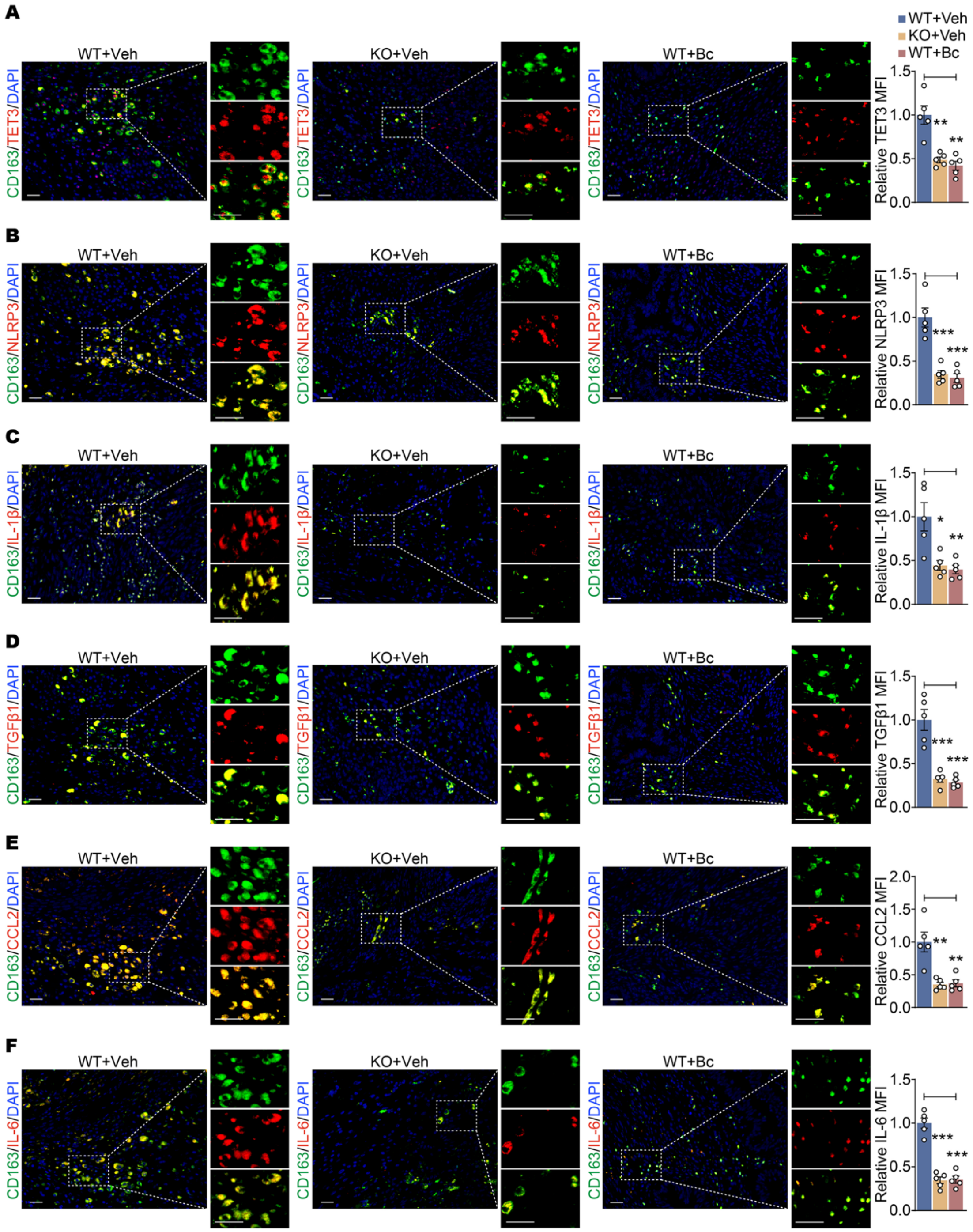

Supplemental Figure 17

(**A**) Representative immunostaining of TET3 (red) in CD163+ macrophages (green) and quantification of macrophage TET3 MFI in endometriosis lesions. (**B**) Representative immunostaining of NLRP3 (red) in CD163+ macrophages (green) and quantification of macrophage NLRP3 MFI in endometriosis lesions. (**C**) Representative immunostaining of IL-1β (red) in CD163+ macrophages (green) and quantification of macrophage IL-1β MFI in endometriosis lesions. (**D**) Representative immunostaining of TGFβ1 (red) in CD163+ macrophages (green) and quantification of macrophage TGFβ1 MFI in endometriosis lesions. (**E**) Representative immunostaining of CCL2 (red) in CD163+ macrophages (green) and quantification of macrophage CCL2 MFI in endometriosis lesions. (**F**) Representative immunostaining of IL-6 (red) in CD163+ macrophages (green) and quantification of macrophage IL-6 MFI in endometriosis lesions. n = 5 mice per genotype. *P < 0.05, **P < 0.01 and ***P < 0.001 indicated the statistic differences from WT+Veh group, by 1-way ANOVA with Tukey's post-test. Scale bar: 50 μm.

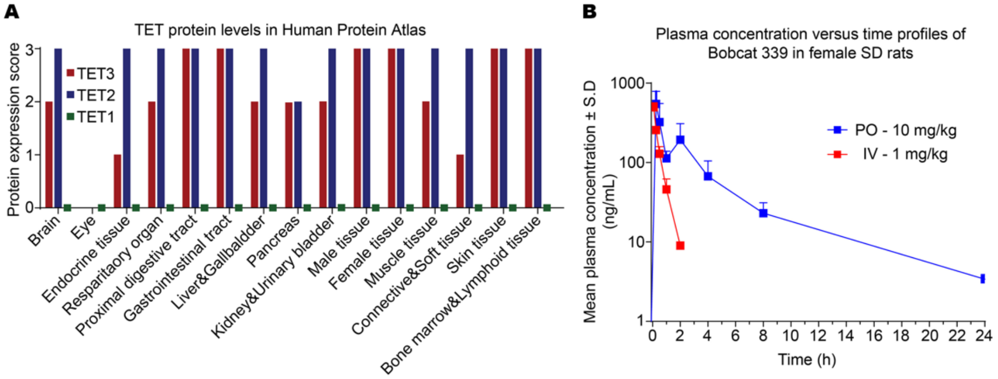

C

Kinetic Solubility	PBS, pH 7.4	>200	µM
Thermodynamic Solubility	PBS, pH 7.4	31	µg/mL
Permeability, MDCK MDR1	MDCK MDR1, Papp A->B	39.8	10^{-6} cm/s
	MDCK MDR1, Papp B->A	48.3	10^{-6} cm/s
Chemical Stability	pH 7.4, 120 min	97	% remaiming
	pH 4.0, 120 min	92	% remaiming
	PH 1.0, 120 min	97	% remaiming
GSH Reactivity	90 min	<1	% disappearance
Blood Stability	Human, 120 min	93	% remaining
	Rat, 120 min	94	% remaining
Plasma Stability	Human, 120 min	96	% remaining
	Rat, 120 min	94	% remaining
Plasma Protein Binding	Human	94	%
	Rat	88.9	%
Liver Microsomes	Human, $t_{1/2}$	>120	min
	Rat, $t_{1/2}$	13	min
Hepatocytes	Human, $t_{1/2}$	369	min
	Rat, $t_{1/2}$	21.8	min
Reactive Metabolite	GSH, HLM, 60 min	No peak	Detection by MS
CYP Inhibition	IC50, CYPs 1A2, 2C9, 2C19, 2D6	>30	µM
	IC50, CYP3A4	>10	µM
CYP3A4 Induction	PXR reporter, 1µM	6	% Rifampicin
	PXR reporter, 10µM	8	% Rifampicin

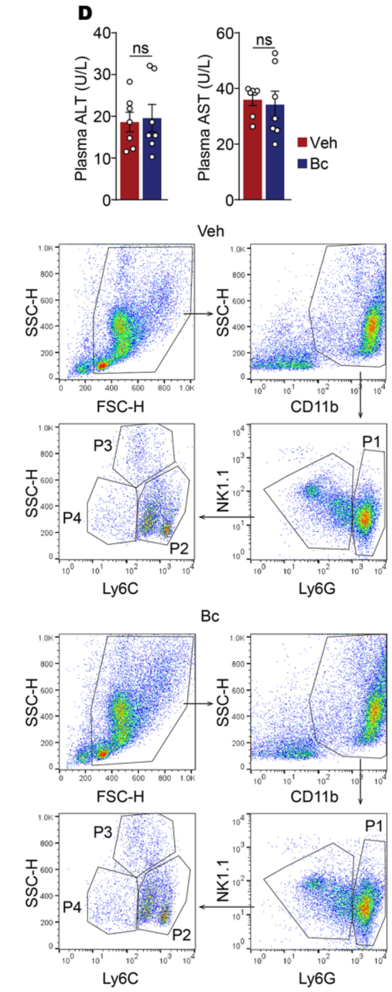

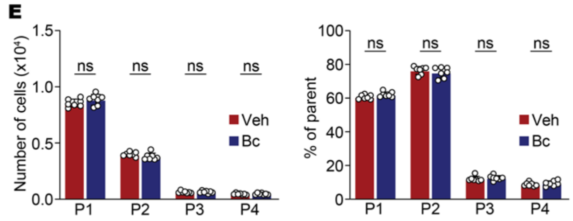

P1: Neutrophils
P2: Ly6C^{high} monocytes and macrophages
P3: Eosinophils
P4: Ly6C^{low} monocytes and macrophages

Supplemental Figure 18

(**A**) Protein levels of TET3, TET2, TET1 in human tissues. Protein expression scores were retrieved from the Human Protein Atlas (data available from v24.0.proteinatlas.org (Tissue expression of TET3 - Summary - The Human Protein Atlas; Tissue expression of TET2 - Summary - The Human Protein Atlas;Tissue expression of TET1 - Summary - The Human Protein Atlas). 3-High expression, 2-Middle expression, 1-Low expression. 0-No expression. (**B**) Plasma concentrations vs. time profiles of Bobcat339 in female SD rats after PO (oral gavage) or IV (tail vein) administration. (**C**) Absorption, distribution, metabolism and excretion (ADME) characteristics of Bobcat339. (**D**) Plasma levels of ALT and AST from WT male mice gavaged with veh or Bobcat339 at 30 mg/kg three times weekly for 7 weeks. (**E**) Representative flow cytometry results of myeloid immune cell populations from bone marrow of mice treated as in **D**. n = 7 mice per group. All data represent the mean ± SEM, ns, no significance, by 2-tailed Student’s t-test.

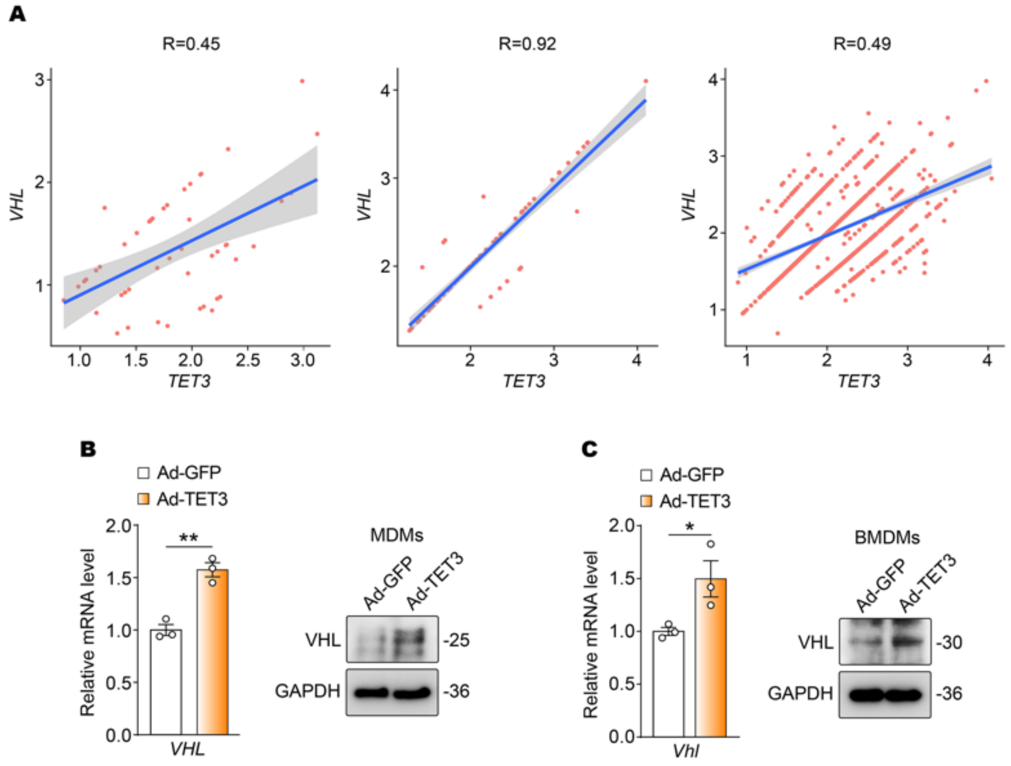

Supplemental Figure 19
(**A**) Scatter plots showing positive expression correlations between *VHL* and *TET3* in macrophages from MASH (left), NSCLC (middle) and endometriosis (right). Refer to Supplemental Table 1 for detailed patient sample information. (**B**) VHL expression in human MDMs infected with Ad-GFP or Ad-TET3 for 24 h, assessed by qRT-PCR (left) and western blot (right). (**C**) Vhl expression in murine BMDMs infected with Ad-GFP or Ad-TET3, assessed by qRT-PCR (left, RNA harvested at 12 h) and western blot (right, protein harvested at 24 h). All data represent the mean ± SEM, *P < 0.05, **P < 0.01, by 2-tailed Student's t-test. Western blot data are representative of two biological repeats.

Supplemental Table 2

**Gene**	**Forward Primer**	**Reverse Primer**
CCL2 (human)	5’-AGTGTCCCAAAGAAGCTGTG-3’	5’-GATTCTTGGGTTGTGGAGTG-3’
NLRP3 (human)	5’-GATCTTCGCTGCGATCAACA-3’	5’-GGGATTCGAAACACGTGCATTA-3’
IL1B (human)	5'-ATGATGGCTTATTACAGTGGCAA-3'	5'-GTCGGAGATTCGTAGCTGGA-3'
TGFB1 (human)	5’-CAAGCAGAGTACACACAGCAT-3’	5’-TGCTCCACTTTTAACTTGAGCC-3’
VHL (human)	5’-GCAGGCGTCGAAGAGTACG-3’	5’-CGGACTGCGATTGCAGAAGA-3’
RPLP0 (human)	5'-GGCGACCTGGAAGTCCAACT-3'	5'-CCATCAGCACCACAGCCTTC-3'
Nlrp3 (mouse)	5'-ATTACCCGCCCGAGAAAGG-3'	5’-TCGCAGCAAAGATCCACACAG-3'
Tgfb1 (mouse)	5’-GGAATACAGGGCTTTCGATT-3’	5’-CTCTGTGGAGCTGAAGCAAT-3’
Vhl (mouse)	5'-CTCAGCCCTACCCGATCTTAC-3'	5'-ACATTGAGGGATGGCACAAAC-3'
Rplp0 (mouse)	5'-GCTCCAAGCAGATGCAGCA-3'	5’-CCGGATGTGAGGCAGCAG-3'
TGFB1 (human ChIP)	5'-CCTGCCGACCCAGCC-3'	5'- CTCGCTGTCTGGCTGCT-3'
NLRP3(human ChIP)	5’-GGGCTGCGACTGCTATAAAT-3’	5’-GGAGCTGGACTTACCCAGATG-3’
IL1B (human ChIP)	5’-GAGTATTGGTGGAAGCTTCTTAGG-3’	5’-CATAGTTTGCTACTCCTTGCCC-3’
CCL2 (human ChIP)	5′-TTTGGTCTCAGCAGTGAATGG-3′	5′-AGTCAAGCAGGAGGAGGGAT-3′
CD274 (human ChIP)	5’-CCCAGCTGCAGCATCTAAGT-3’	5’-AGGCCAAGGTCAATGTGTCT-3’
