# Supplementary figures and images for: TET3 is a common epigenetic immunomodulator of pathogenic macrophages
Source: J Clin Invest. 2025 Aug 12;135(21):e194879. doi: 10.1172/JCI194879 (PMC12578387; doi:10.1172/JCI194879)

Figure 2

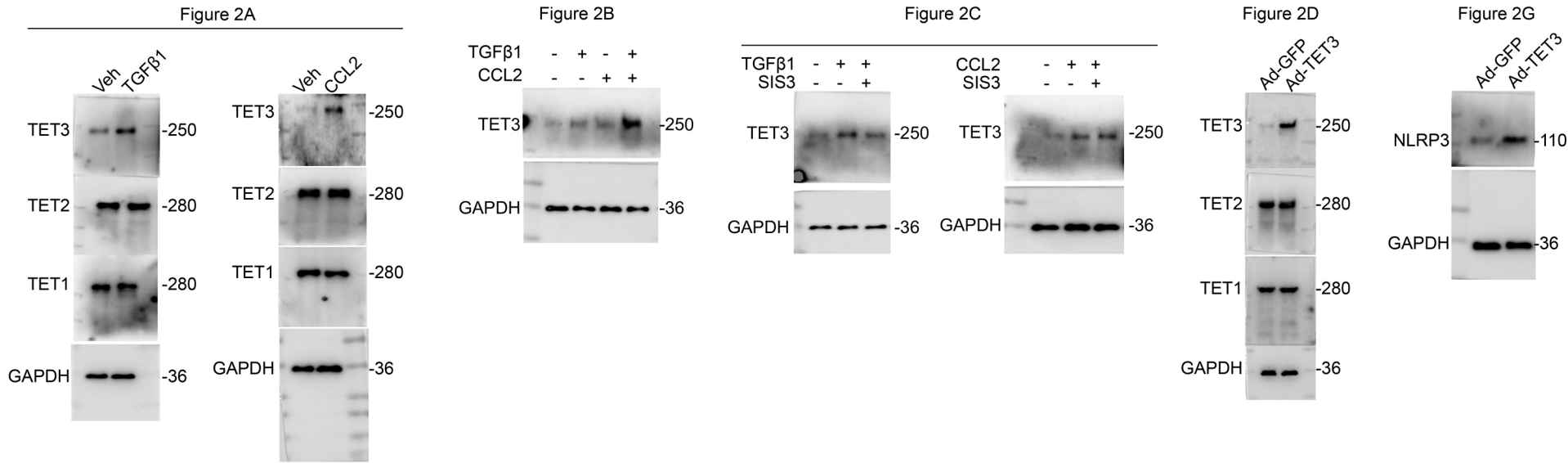

Figure 3

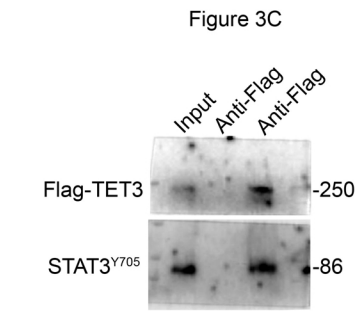

Figure 4

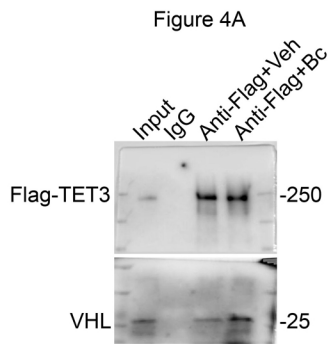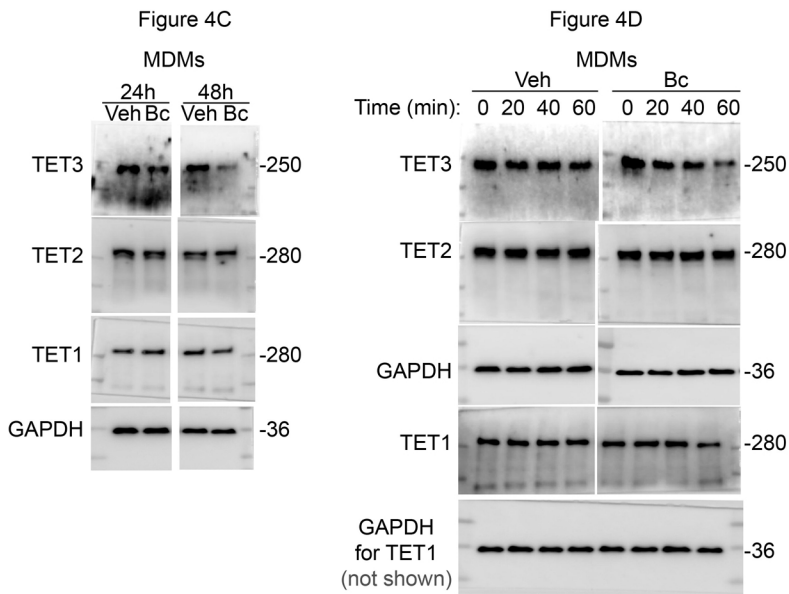

Figure 6

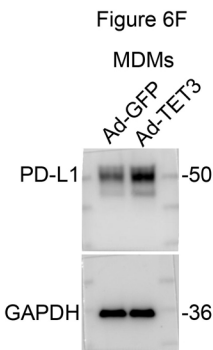

Figure S3

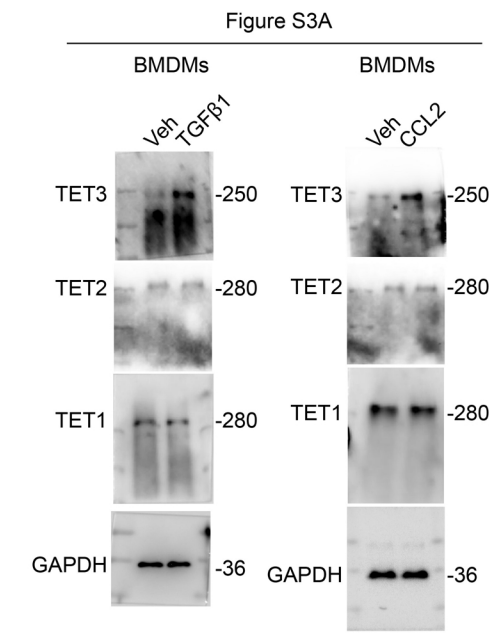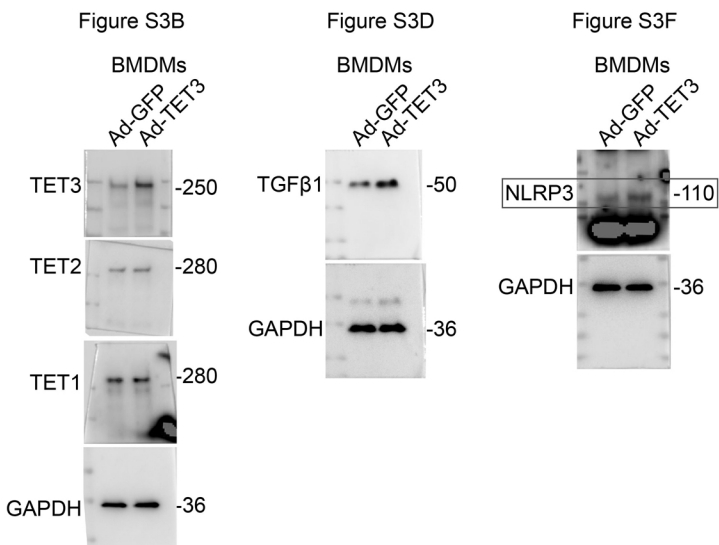

Figure S3D

Figure S3F

Figure S6

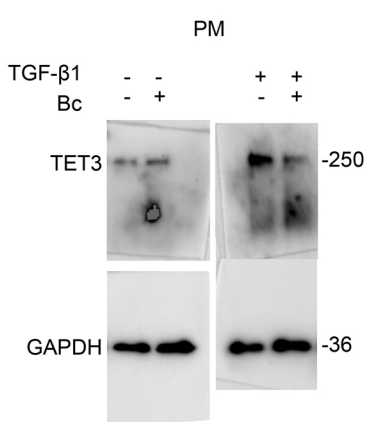

Figure S7

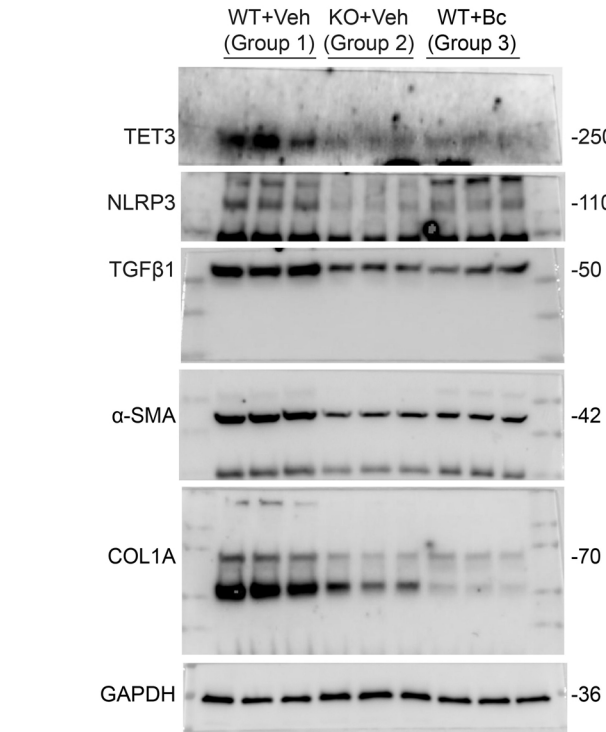

Figure S19

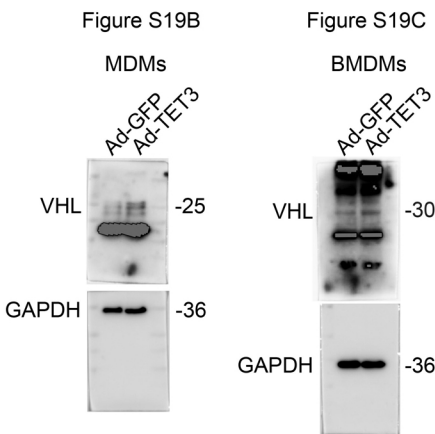

Figure S19C

Supplement: Unedited blot and gel images [file jci-135-194879-s295.pdf]
